# Automatic molecular fragmentation by evolutionary optimisation

**DOI:** 10.1186/s13321-024-00896-z

**Published:** 2024-08-19

**Authors:** Fiona C. Y. Yu, Jorge L. Gálvez Vallejo, Giuseppe M. J. Barca

**Affiliations:** 1 School of Computing, Australian National University, Canberra, 2601 ACT Australia; 2 School of Computing and Information Technology, The University of Melbourne, Melbourne, 3052 VIC Australia

**Keywords:** Molecular fragmentation, Quantum chemical calculations, Many body expansion, Fragment molecular orbital, Molecular graph theory

## Abstract

Molecular fragmentation is an effective suite of approaches to reduce the formal computational complexity of quantum chemistry calculations while enhancing their algorithmic parallelisability. However, the practical applicability of fragmentation techniques remains hindered by a dearth of automation and effective metrics to assess the quality of a fragmentation scheme. In this article, we present the Quick Fragmentation via Automated Genetic Search (QFRAGS), a novel automated fragmentation algorithm that uses a genetic optimisation procedure to generate molecular fragments that yield low energy errors when adopted in Many Body Expansions (MBEs). Benchmark testing of QFRAGS on protein systems with less than 500 atoms, using two-body (MBE2) and three-body (MBE3) MBE calculations at the HF/6-31G* level, reveals mean absolute energy errors (MAEE) of 20.6 and 2.2 kJ $\hbox {mol}^{-1}$, respectively. For larger protein systems exceeding 500 atoms, MAEEs are 181.5 kJ $\hbox {mol}^{-1}$ for MBE2 and 24.3 kJ $\hbox {mol}^{-1}$ for MBE3. Furthermore, when compared to three manual fragmentation schemes on a 40-protein dataset, using both MBE and Fragment Molecular Orbital techniques, QFRAGS achieves comparable or often lower MAEEs. When applied to a 10-lipoglycan/glycolipid dataset, MAEs of 7.9 and 0.3 kJ $\hbox {mol}^{-1}$ were observed at the MBE2 and MBE3 levels, respectively.

**Scientific Contribution** This Article presents the Quick Fragmentation via Automated Genetic Search (QFRAGS), an innovative molecular fragmentation algorithm that significantly improves upon existing molecular fragmentation approaches by specifically addressing their lack of automation and effective fragmentation quality metrics. With an evolutionary optimisation strategy, QFRAGS actively pursues high quality fragments, generating fragmentation schemes that exhibit minimal energy errors on systems with hundreds to thousands of atoms. The advent of QFRAGS represents a significant advancement in molecular fragmentation, greatly improving the accessibility and computational feasibility of accurate quantum chemistry calculations.

**Supplementary Information:**

The online version contains supplementary material available at 10.1186/s13321-024-00896-z.

## Introduction

In contemporary research within the fields of drug discovery, synthetic biology, chemistry, and materials science, a significant challenge is the limited ability to accurately model large-scale molecular processes using computational methods. Notable examples of this include the computational study of carbon capture and sequestration using porous materials [21, 62], the accurate modelling of interactions between ligands and proteins for effective drug design [1, 12, 56], and the simulation of the degradation or removal of organic waste materials, such as pesticides, using novel catalysts [25, 40]. These problems, along with many others, necessitate chemically accurate models of molecular systems including explicitly hundreds to thousands of atoms.

Quantum chemistry (QC) calculations have the potential to provide such models. However, the computational time required by accurate *ab initio* QC methods increases extremely fast-formally faster than ${\mathcal {O}}(N^4)$ [6, 36, 61]-with the size of the system. This rapid growth in computational demand severely limits the applicability of these methods to large molecular systems. Additionally, the algorithms fundamental to QC calculations are generally not optimised to leverage the extensive parallelism inherent in contemporary supercomputer architectures, which further complicates this challenge.

Molecular fragmentation is an effective strategy for tackling scalability and parallelisation issues in quantum chemical modelling. This suite of methodologies is based on the premise that quantum chemical interactions are sufficiently localised, allowing a chemical system to be divided into smaller segments known as monomers. To approximate the energy of the entire, unfragmented system, fragmentation approaches incrementally include the effects of larger fragments. These fragments, which encompass interactions among monomers, range from dimers and trimers to larger *n*-mers.

For example, in fragmentation methods based on the Many Body Expansion (MBE) [53], the energy of the system is obtained as the following sum over fragments

1 $$\begin{aligned} E_{MBE} = \sum _I E_I + \sum _{I<J} \Delta E_{IJ} + \sum _{I<J<K} \Delta E_{IJK} + ... \end{aligned}$$

where $E_I$ is the energy of monomer *I*, $\Delta E_{IJ}$ and $\Delta E_{IJK}$ are dimer and trimer energy corrections defined as follows

2 $$\begin{aligned} \Delta E_{IJ}= & {} E_{IJ} - E_I - E_J, \end{aligned}$$

3 $$\begin{aligned} \Delta E_{IJK}= & {} E_{IJK}- \Delta E_{IJ} - \Delta E_{IK} - \Delta E_{JK}\nonumber \\{} & {} - E_I - E_J - E_K \end{aligned}$$

where $E_{IJ}$ is the energy of a dimer system obtained as the union of monomers *I* and *J*, and $E_{IJK}$ is the energy of a trimer system obtained as the union of monomers *I*, *J*, *K*.

The calculations of two-body and higher order terms ($\Delta E_{IJ}$, $\Delta E_{IJK}$, etc.) are only performed on fragments that are spatially close together, yielding an asymptotic scaling of ${\mathcal {O}}(N)$ with system size [16, 44].

The hierarchical nature of the MBE allows it to approximate the total energy to greater accuracy through the systematic inclusion of higher order terms [57]. In addition, the energy calculations of the many-body fragments (monomers, dimers, etc.) can be performed independently, thereby exposing significant opportunities for exploiting large-scale parallelism [16].

Although fragmentation methods offer considerable advantages, they are usually not applicable in a general black-box fashion to medium and large molecular systems. This can be primarily attributed to a dearth of automated fragmentation procedures. Currently, the design of fragments that yield accurate results is typically performed manually, requiring a laborious iterative combination of chemical intuition and trial and error. This not only limits the size of systems that can be accurately fragmented and studied, but also renders the resulting fragmentation schemes largely nontransferable across molecular systems and application studies.

Automated bond-breaking fragmentation algorithms have been developed in conjunction with the Molecular Tailoring Approach [26, 37], Systematic Molecular Fragmentation [14, 15, 20], and the Generalised Energy Based Fragmentation [31, 32, 42]. These techniques create fragments from small units like functional groups or non-hydrogen atoms, selecting a specific size based on distance criteria (either the number of bonds or spatial distance). However, these fragmentation algorithms generally do not explicitly consider the surrounding chemical environment nor the energetic effects of the bond breaking, as fragment generation is primarily guided by basic distance and connectivity factors.

The main challenge in constructing a high quality fragmentation scheme is the generation of an optimal set of fragments that minimises the fragmentation energy error while retaining a user-defined fragment size. This goal is elusive and remains largely unaccomplished, primarily due to the absence of fragmentation strategies focused on generating high-quality fragments. A key issue with current schemes is their lack of explicit consideration for the types of bonds being broken. It is well-recognised that different sets of fragments, resulting from breaking various bonds, can lead to varied approximations of the final energy value. The severance of different bonds, yielding unique sets of fragments, results in the loss of distinct chemical interactions. This, in turn, leads to different estimates of the final energy of the unfragmented system.

**Fig. 1:**
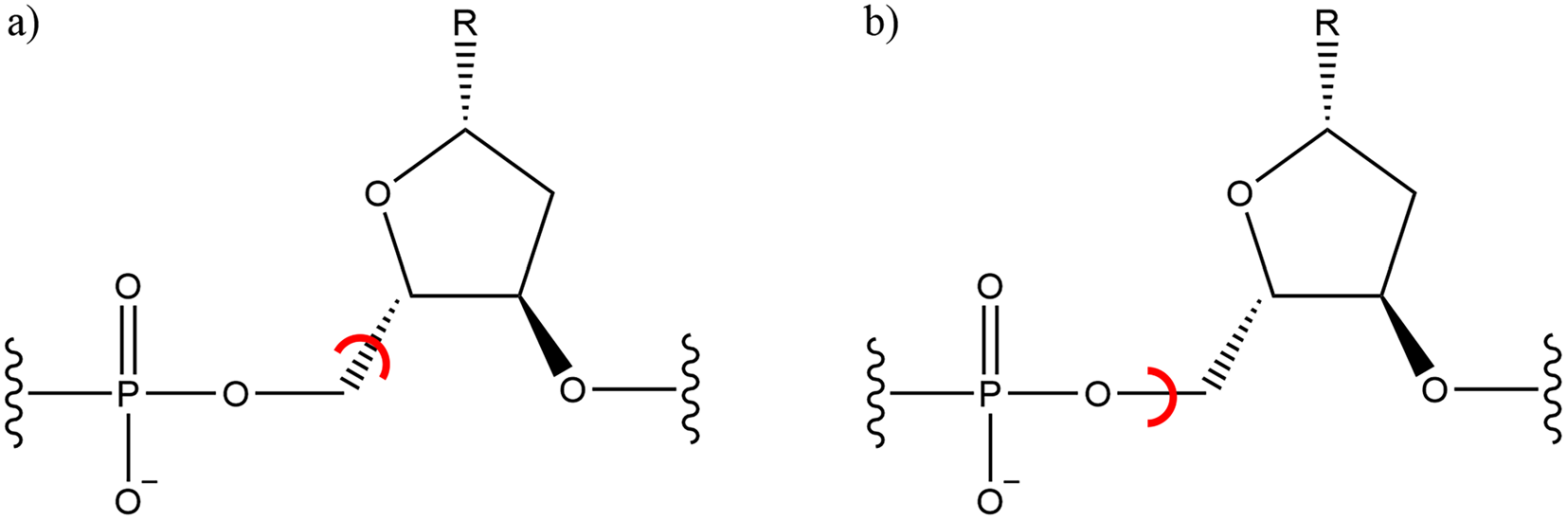
The two alternative fragmentation schemes for fragmenting DNA used in [48]. Fragments are formed by either **a** breaking the C–C bond or **b** breaking the C–O bond

The significance of the nature of bond breaking in molecular fragmentation was highlighted, for example, in a previous study which focused on the application of the Fragment Molecular Orbital (FMO) [22, 38] method to DNA molecules. In this study, DNA was fragmented by cutting either the carbon-carbon (C-C) or carbon-oxygen (C-O) bond between the five-carbon sugar and phosphate group, as shown in Fig. 1. Considering the close proximity of these bonds, one might anticipate similar energy estimations for the intact system using both fragmentation schemes. However, the calculated energies for the two approaches showed a significant difference, exceeding 18 kJ $\hbox {mol}^{-1}$ [48]. It is noteworthy that these fragmentation errors scale linearly with the fragment count.

Thus, the issue at hand raises an important question: *How can we measure the effectiveness of a molecular fragmentation scheme?*

In current methodologies, the efficiency of these schemes is not known beforehand. Instead, their effectiveness is only determined retrospectively. This is done by calculating the system energy with and without fragmentation and comparing the results. However, for large molecular systems comprising hundreds to thousands of atoms, calculating the unfragmented system energy with traditional QC approaches is impractically demanding. The primary aim of fragmentation methods is, in fact, to circumvent this very challenge. Consequently, employing such a metric for evaluation is neither practical nor reasonable, and we necessitate the development of a more feasible alternative approach.

In this Article, we present a novel automatic fragmentation scheme that aims to obtain the optimal sets of fragments for a molecular system. This new approach, named the Quick Fragmentation via Automated Genetic Search (QFRAGS), employs a specialised scoring function to assess fragmentation quality. The scoring function is designed and parameterised to obtain a strong correlation with the energy error of the resulting fragmentation scheme, thereby circumventing the usage of an expensive and generally unusable direct energy error metric. This enables recasting the fragmentation problem as an evolutionary optimisation of the scoring function, which is a rapid, cost-efficient and accurate process.

We begin in Sect. Materials and methods with describing the datasets of molecular systems QFRAGS was applied to. This is followed by a description of the methodology of the fragmentation scheme, particularly, the scoring function used to describe the quality of fragmentation and the mathematical representation of the molecular system and its fragmentation. Then, we detail the approach used for the optimisation of weights in the scoring function. Next, in Sect. Algorithms, we discuss the algorithms utilised for the optimisation of the scoring function. The optimised weights are reported in Sect. Results and discussion and these were used in the application of QFRAGS to over 1000 protein systems. The corresponding fragment sizes generated and their corresponding energetic accuracy are discussed in Sect. Results and discussion. To further exemplify the accuracy of QFRAGS, a comparison to three manual fragmentation schemes is presented in Sect. Results and discussion. Sect. Conclusion concludes.

## Materials and methods

### Datasets

The automatic fragmentation algorithm will be applied across a range of biologically significant protein systems. These systems hold special relevance in the fields of drug design and synthetic biology, which are prominent areas of application for molecular fragmentation techniques.

**Fig. 2:**
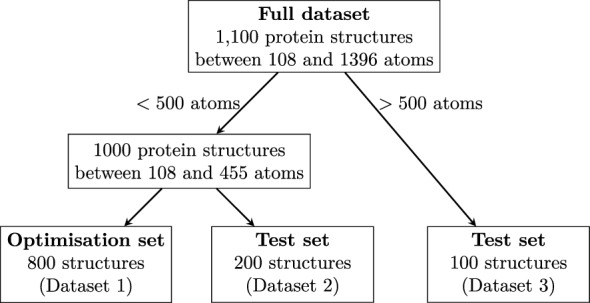
Classification of datasets used in this study

Figure 2 shows the classification of the three protein datasets used in this study. Cumulatively, the datasets comprise 1100 protein structures obtained from two different sources. A subset of 100 protein structures, included in Dataset 3 as indicated in Fig. 2, was extracted from the PDB-Bind database [64]. These structures are characterised by having more than 500 atoms each. Notably, protein systems with less than 500 atoms are rare in the PDB-Bind dataset. To address the shortage of such systems, we took existing protein structures in the PDB-Bind dataset and fragmented these to generate 1000 additional systems with less than 500 atoms. This involved severing single ${\hbox {C}_\alpha }$-N or $\hbox {C}_\alpha$-C bonds and valence is restored by appending hydrogens along the axis of the bond cut. Specifically, the coordinates of the hydrogen cap ${\textbf{x}}(H)$ is given by

4 $$\begin{aligned} {\textbf{x}}(H) = {\textbf{x}}(i) + \frac{r(i) + r(H)}{r(i) + r(j)}\left( {\textbf{x}}(j) - {\textbf{x}}(i)\right) \end{aligned}$$

where ${\textbf{x}}$ denotes a Cartesian coordinate, *r* is the standard covalent radius given by Cordero *et al* [18], and *i*, *j* denote the atoms belonging to the severed bond.

None of the resulting 1000 systems were derived from structures present in Dataset 3. All datasets are mutually exclusive.

All protein structures were hydrogenated using the PDBFixer software at the default pH of 7.0. All protein structures herein comprise one polypeptide chain and no metal-dependent structures are present within the dataset.

**Fig. 3:**
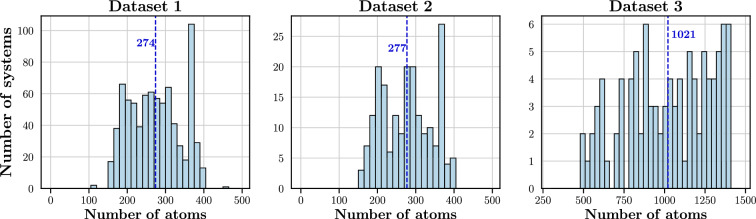
Size distribution of systems belonging to the three datasets: Dataset 1, 2 and 3. Averages are indicated by vertical lines and the corresponding values are reported.

As illustrated in Fig. 2, the classification of the datasets involves an initial split of the full dataset based on the size where 500 atoms is the threshold. This threshold distinguishes between structures taken directly from the PDB-Bind dataset as opposed to the generated structures. The dataset of 1000 generated systems is further divided in two datasets with a 80:20 split. Here, 80% of the structures are used for the optimisation of hyperparameters within the fragmentation algorithm (Dataset 1) and 20% is used to test the application of QFRAGS with the optimised hyperparameters (Dataset 2). The optimisation of the hyperparameters is presented in Sect. 2.4. Similar to Dataset 2, Dataset 3 is also a test dataset but for protein systems with more than 500 atoms. The size distributions of the three datasets are shown in Fig. 3.

Structures used for the optimisation of hyperparameters (Dataset 1) comprise a diverse set of protein sequences; 90.4% and 3.3% of protein pairs exhibited pairwise sequence identity (PID) scores below 20% and between 20% and 30%. On the other hand, 6.3% of structure pairs exhibit PID values greater than 30%. Furthermore, these structures also exhibit a wide range of functionalities including: signalling proteins, structural proteins, toxins, viral proteins, enzymes, DNA/RNA binding proteins, transcription and transport proteins.

In addition, we also applied QFRAGS to 10 glycolipid or lipoglycans systems ranging between 368 and 727 atoms to demonstrate its applicability to systems beyond proteins. All structures were taken from the Human Metabolome Database (HMDB) directly. These structures were selected on the basis of size. Specifically, structures within HMDB were sorted against size and we randomly selected 10 systems belonging in the top 20 largest glycolipids/lipoglycans. We selected glycolipids and lipoglycans structures to apply QFRAGS to for two main reasons. Firstly, such systems are of particular biological significance as they include structures that form part of cell membranes responsible for structural integrity or modulating signal transduction events, as well as serving as intermediates in the synthesis pathway of glycans where disruptions can lead to congenital disorders of glycosylation. Secondly, unlike proteins which have an intuitive monomeric unit (amino acids), lipoglycans/glycolipids do not and we endeavour to examine the performance of QFRAGS on such systems.

### Single point Hartree-Fock energy calculations

In this study, single point energy calculations on molecular systems are conducted to evaluate the effectiveness of the proposed fragmentation algorithm and to fine-tune the hyperparameters utilised in the process. These calculations were consistently carried out at the Hartree-Fock theoretical level, employing the 6-31G* basis set. Unless specifically indicated, all computations were executed using the Extreme-scale Electronic Structure System (EXESS) quantum chemical software package [7–9, 27, 50, 59, 60].

To assess the accuracy of the proposed fragmentation scheme, we use the difference between the energy of the unfragmented system ($E_{tot}$) and the energy obtained via fragmentation ($E_{f}$):

5 $$\begin{aligned} \Delta E = E_{tot} - E_{f}. \end{aligned}$$

Evaluating $\Delta E$ requires performing full system energy calculations ($E_{tot}$) on datasets of protein systems, each containing hundreds or thousands of atoms. Prior research has underscored the limitations of the traditional Superposition of Atomic Densities (SAD) as an initial guess approach [5]. Notably, SAD initial guesses often face convergence challenges in systems comprising hundreds of atoms [29]. Additionally, SAD density matrices are typically charge neutral [41], which is usually not compatible with protein systems, as they frequently contain charged chemical groups. Consequently, due to the convergence issues encountered when applying SAD to large systems with charged groups, this study adopts an alternative initial guess strategy for full system calculations.

Specifically, our initial guess starts by dividing the molecular system into monomers, each containing approximately 30 atoms. Only single bonds are severed here. *Ab initio* calculations are then performed on each monomer where SAD is used. Once the monomer densities converge, they are combined to form a block-diagonal density matrix, which is used as the initial guess for the full system calculation.

The fragmentation-based single point energy calculations ($E_{f}$) were performed using two methods: the Many Body Expansion (MBE) (Eq. (1)) and the Fragment Molecular Orbital (FMO) approach, both at the dimer (MBE2 and FMO2) and trimer (MBE3 and FMO3) levels. FMO is similar to MBE in that it utilises Eq. (1) to recombine fragment energies. However, rather than performing fragment energy calculations *in vacuo* as in MBE, in FMO these are performed in a self-consistent manner with respect to an electrostatic embedding, known also as Coulomb bath or ESP (electrostatic potential), of the surrounding monomers [38]. Furthermore, in the MBE implementation, hydrogen capping is used to restore valence at the sites of bond breaking. Hydrogen atoms are appended to fragments along the axis of the broken bond. On the other hand for FMO, the adaptive frozen orbital (AFO) approach was employed for the treatment of broken bonds. This involves freezing the molecular orbital of the broken bond [23, 24]. All FMO calculations were performed using the GAMESS quantum chemical software package [10].

In dimer calculations, all possible dimers were included and for trimer calculations, all possible dimers and trimers were included.

### Methods for automatic fragmentation

In this section, we delineate the methods underpinning our proposed automatic fragmentation algorithm. We detail the representation of a molecular system, elucidate the metrics employed to evaluate the quality of fragmentation, and explain the representation of fragmentation involving bond breaking.

#### Molecular graph characterisation

**Fig. 4:**
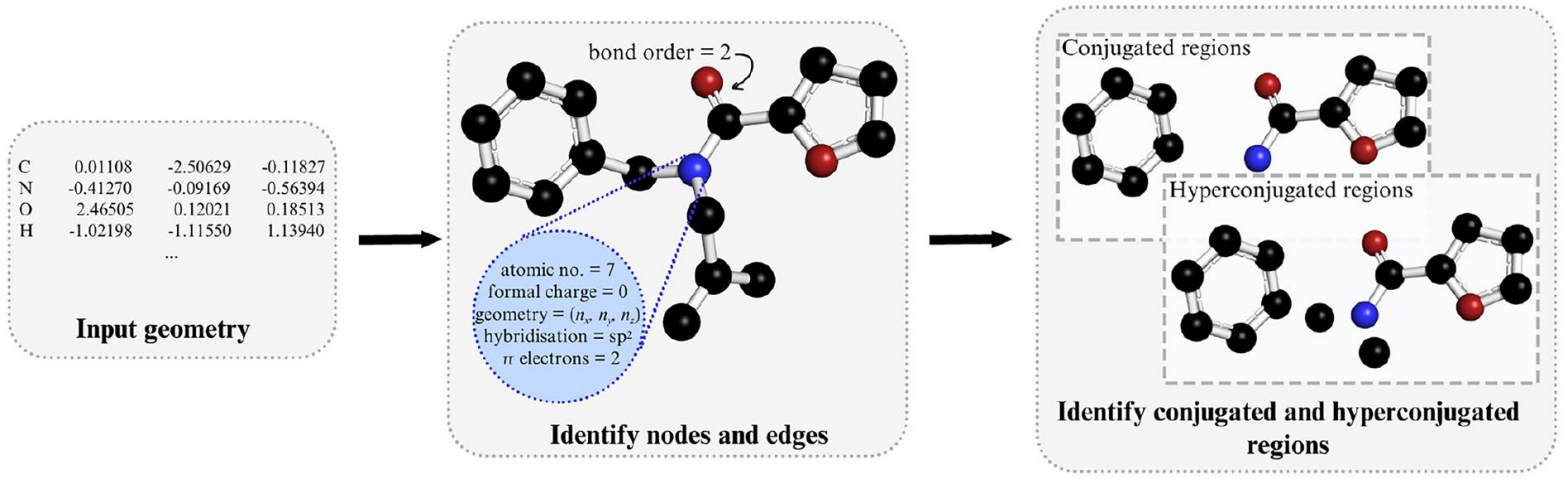
Graph representation of an input molecular system. Hydrogen atoms are omitted for clarity. Loner nodes in hyperconjugated regions correspond to hyperconjugated donor/acceptor C-H

As illustrated in Fig. 4, in our fragmentation algorithm, a molecular system is represented as a graph where the nodes and edges correspond to atoms and covalent bonds, respectively. Similar representations have been employed across multiple studies that use graphs to capture the connectivity of molecular systems [3, 4, 11].

In the molecular graph representation, nodes and edges are assigned distinct attributes to accurately map the molecular system. Each node, representing an atom, is characterised by several attributes: the atomic number, formal charge, number of $\pi$ electrons, hybridisation state, and its Cartesian coordinates. The attribute for the number of $\pi$ electrons holds a non-zero value only for atoms that are components of a conjugated system. In such cases, this value corresponds to the number of $\pi$ electrons that the atom contributes to the system. For instance, the nitrogen atom in a pyrrole molecule possesses two $\pi$ electrons.

In contrast, bond order represents the sole attribute of an edge. Edges, or bonds, along with their respective bond orders, are determined based on the distances found in the Computational Chemistry Comparison and Benchmark DataBase, which includes experimental bond lengths [54].

The program not only characterises the nodes and edges in the molecular graph but also identifies regions of conjugation and hyperconjugation. This identification is crucial for understanding how fragmentation might disrupt these molecular features. However, aromatic regions are not considered in this context, as the current implementation is limited to the breaking of single bonds. The rationale behind this limitation is discussed in greater detail in Sect. 3.1.1.

Conjugated regions identified as part of the molecular graph refer to groups of atoms exhibiting $\pi$-conjugation. This effect occurs when there are alternating single and double/triple bonds along a chain of the structure [46], and $\pi$-electrons across the atoms becoming delocalised. To represent this, in the program, a conjugated group is defined as a group of connected nodes where every node has a hybridisation state of either $\hbox {sp}^2$ or sp.

Hyperconjugation involves the interaction between polarised $\sigma$-bonds and nearby $\pi$-orbitals [2]. $\pi$-orbitals are found in various forms, including conjugated systems, double or triple bonds, and lone pairs on atoms. Polarised $\sigma$-bonds are typically of the form C-X, where X is a hydrogen or a halogen. In a hyperconjugated pair, there are donor and acceptor groups, which can be either $\pi$-systems or $\sigma$-systems, or a combination of both. However, for a pair to be considered hyperconjugated, it must include one $\sigma$-system and one $\pi$-system. In the current software implementation, the donor and acceptor groups are limited to being at most three bonds apart. Table 1 outlines the specific hyperconjugation donor and acceptor groups identified in this scheme. The conjugated groups that are identified serve as potential donors and acceptors for hyperconjugation.

**Table 1:** Classification of hyperconjugated groups

Group	Type	Classification
C=C	$\pi$	Donor/acceptor
C$\equiv$C	$\pi$	Donor/acceptor
C-H	$\sigma$	Donor/acceptor
C=O	$\pi$	Acceptor
C-F	$\sigma$	Acceptor
C-Cl	$\sigma$	Acceptor
C-Br	$\sigma$	Acceptor
C-I	$\sigma$	Acceptor
$\hbox {C}^{+}$($\hbox {sp}^{2}$)	$\pi$	Acceptor
$\hbox {C}^{-}$($\hbox {sp}^{2}$)	$\pi$	Donor
N($\hbox {sp}^{3}$)	$\pi$	Donor
O($\hbox {sp}^{3}$)	$\pi$	Donor

Corresponding $\sigma$/$\pi$ nature that the program identifies. Hybridisation states and charges are shown on relevant atoms

#### Scoring function

In pursuit of an alternative non-energy-based metric to describe the quality of fragmentation, we employ the following scoring function

6 $$\begin{aligned} s= & {} \beta _{1} p_{pe} + \beta _{2} p_{conj} + \beta _{3} p_{hyper} +\beta _{4} p_{vol} \nonumber \\{} & {} + \beta _{5} p_{comp} + \beta _{6} p_{vrange} \end{aligned}$$

where the penalty factors $p_i$ are designed to account for various chemical and implementation factors. Broadly, these penalty factors fall into two categories. The first category encompasses penalties related to potential energy, conjugation, and hyperconjugation, with the primary objective of maintaining the chemical environment’s integrity. The second category focuses on managing fragment size. This includes penalties based on the volume of fragments, the number of fragments, and the range of their volumes, ensuring that the fragments produced closely match the desired target size.

Each penalty $p_i$ is a function that takes a set of broken bonds as input and produces a corresponding penalty value associated with the factor *i*. The parameters $\beta _i$ serve as the weights for these penalties $p_i$. The subsequent text outlines the formulation of each penalty term.

***Potential energy penalty***

The $p_{pe}$ penalty is a measure of the change in the potential energy of the system induced by the fragmentation scheme. This is evaluated according to the formula:

7 $$\begin{aligned} p_{pe}= & {} \frac{1}{1+\exp {(-\frac{\lambda }{\gamma }(\Delta _{pe} - \gamma d))}}\nonumber \\{} & {} + \frac{1}{1+\exp {(-\frac{\lambda }{\gamma }(-\Delta _{pe} - \gamma d))}}. \end{aligned}$$

The formula for $p_{pe}$ comprises two logistic sigmoid functions that are mirror images of each other and handle positive and negative $\Delta _{pe}$ values. The $\Delta _{pe}$ term is the difference between the energy of the total unfragmented system ($E_{tot}$) and the total energy ($E_{MBE1}$) obtained at the one-body MBE level (MBE1)

8 $$\begin{aligned} \Delta _{pe} = E_{tot} - E_{M\,BE1}. \end{aligned}$$

Here, both the $E_{tot}$ and $E_{MBE1}$ energies are calculated using the universal force field (UFF) [43, 52]. This was selected due to its accessibility with parameters available for all atoms in the periodic table [35] as well as its low computational evaluation time.

The values of the parameters $\lambda$ and *d* in Eq. (7) are 1.963 and 6, respectively. These are defined based on where the sigmoid function has sufficiently approached the lower and upper asymptotes. The definition of “sufficiently approached” used is that reported by McDowall and co-workers [45] where the function is considered to have sufficiently approached the asymptote when it is 5% above or below it. Thus, $\lambda$ and *d* were selected by setting the lower and upper threshold values of the positive sigmoid function to correspond to $\Delta _{pe} = 10$ and $\Delta _{pe} = 40$ kJ $\hbox {mol}^{-1}$, respectively. We call these $\Delta _{pe}$ values the boundary points.

The $\gamma$ parameter in Eq. (7) is a scaling function and is defined as follows

9 $$\begin{aligned} \gamma = \frac{\sqrt{N_f} \cdot N_A^{min}}{n_{t}} \end{aligned}$$

where $N_f$ is the number of fragments, $N_A^{min}$ is the number of atoms in the smallest fragment, and $n_t$ is the target fragment size. The role of this scaling factor is to modulate the range between the boundary points. If $\gamma$ is small, the range becomes narrower and the opposite is true for larger $\gamma$ values.

The UFF employed in the computation of $p_{pe}$ implements simple functional forms, and may face difficulty in accounting for effects such as conjugation and electronic effects including hyperconjugation [13, 66]. Thus, the effects of the fragmentation on conjugation and hyperconjugation are included as separate penalties in the scoring function.

***Conjugation penalty***

The conjugation penalty ($p_{conj}$) is defined as

10 $$\begin{aligned} p_{con\,j} = \frac{1}{N_{cs}}\sum _k^{N_{cs}}{\mathcal {S}}\left( \Delta _{conj}^k \right) \end{aligned}$$

where *k* indexes the conjugated systems that have been disrupted by fragmentation, $N_{cs}$ is the total number of affected conjugated systems, ${\mathcal {S}}$ is a normalisation function, $\Delta _{conj}^k$ factors for conjugated system *k* and is defined as follows

11 $$\begin{aligned} \Delta _{con\,j} = \frac{1}{cs}\left( \frac{1}{N_{A}} \sum _i^{N_{A}} \frac{N_{e}^{i}}{N_{A}^{i}} - cs\right) . \end{aligned}$$

Here, $N_{A}$ is the number of atoms within the conjugated system, with each atom being indexed by *i*. The term $N_{e}^{i}$ denotes the number of $\pi$ electrons contributed by atom *i* to the conjugated system. Additionally, ${N_{A}^{i}}$ refers to the aggregate count of atoms in the conjugated system that remains interconnected after fragmentation, specifically in the fragment to which atom *i* pertains. The terms *cs* is a conjugation score of the system given by

12 $$\begin{aligned} cs = \frac{1}{N_{A}} \sum _i^{N_{A}} \frac{N_e^{i}}{N_{A}} \end{aligned}$$

For example, consider the case of pyrrole which consists of one conjugated system ($N_{cs} = 1$). In pyrrole, all non-hydrogen atoms participate in conjugation ($N_{A} = 5$). Each carbon atom contributes one $\pi$ electron and the nitrogen atom contributes two $\pi$ electrons from its lone pair. Thus, the conjugation score of pyrrole is

13 $$\begin{aligned} cs_{pyrrole} = \frac{1}{5} \left( \frac{1}{5} + \frac{1}{5} + \frac{1}{5}+ \frac{1}{5} + \frac{2}{5} \right) = \frac{6}{25} \end{aligned}$$

The normalisation function ${\mathcal {S}}$ in Eq. (10) has the form

14 $$\begin{aligned} {\mathcal {S}}(\Delta _{conj}) = \frac{1-\exp {(-\lambda \Delta _{conj})}}{1+\exp {(-\lambda \Delta _{conj})}} \end{aligned}$$

Similar to the normalisation function for the potential energy, the exponent $\lambda$ is chosen by setting a boundary value to correspond to 5% below the upper asymptote. Specifically, we set ${\mathcal {S}}(\Delta _{conj}^{max}) = 0.95$ where $\Delta _{conj}^{max}$ is the maximum possible value of $\Delta _{conj}$ and arises when every bond in the conjugated system is broken. The value of $\Delta _{conj}^{max}$ is obtained as follows

15 $$\begin{aligned} \Delta _{conj}^{max} = N_{A} - 1 \end{aligned}$$

***Hyperconjugation penalty***

The hyperconjugation penalty $p_{hyper}$ is calculated using the following formula

16 $$\begin{aligned} p_{hyper} = \frac{1}{N_{hs}} \sum _k^{N_{hs}} \frac{1}{\gamma _k}{\mathcal {S}}\left( \Delta _{hyper}^{k} \right) . \end{aligned}$$

Here *k* indexes the hyperconjugated systems, each comprising a donor and an acceptor group, disrupted by fragmentation. The $N_{hs}$ term represents the total count of these affected systems, $\gamma _k$ is the bond count between the donor and acceptor in system *k*, ${\mathcal {S}}$ is a normalisation function, and $\Delta _{hyper}^{k}$ is defined for each hyperconjugated pair *k* as follows

17 $$\begin{aligned} \Delta _{hyper} = \frac{1}{N_{d}} \sum _i^{N_{d}} \frac{N_{e}^{i}}{N_{A}^{i}} - \frac{1}{N_{a}} \sum _j^{N_{a}}\frac{N_{e}^{j}}{N_{A}^{j}} \end{aligned}$$

Eq. (17) captures the change in electron distribution across the donor and acceptor atoms due to fragmentation. In the first term, $N_{d}$ denotes the number of fragments containing donor atoms from the hyperconjugated pair. The index $i$ identifies these fragments. $N_{e}^i$ and $N_{A}^i$ respectively represent the number of electrons donated and the count of atoms in fragment $i$. The second term mirrors the first, focusing on acceptor atoms. $N_{a}$ indicates the count of fragments with acceptor atoms, with $j$ indexing these fragments. $N_{e}^j$ and $N_{A}^j$ respectively represent the number of electrons accepted and the count of atoms in fragment $j$.

**Fig. 5:**
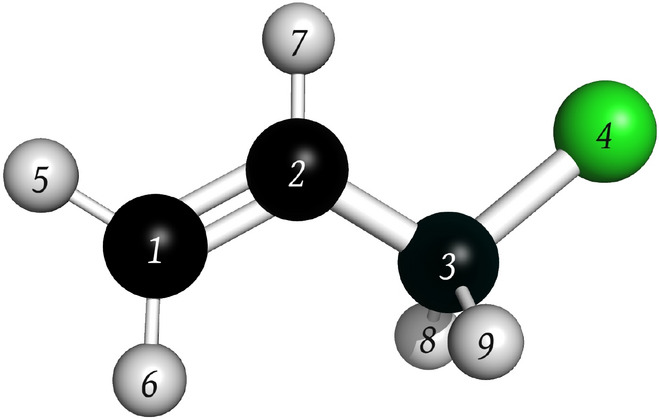
Labelled ball and stick model of 3-chloroprop-1-ene. Green, black, white spheres correspond to chloride, carbon and hydrogen atoms, respectively

To illustrate the meaning of the terms within Eq. (17), consider fragmenting the molecule shown in Fig. 5 by cutting the bond between atoms 2 and 3. There is only one hyperconjugation pair present, where the donor is the C=C bond (atoms 1 and 2) and the acceptor is the C-Cl bond (atoms 3 and 4). After fragmentation, the two donor atoms remain connected, therefore in the first term of Eq. (17), $N_{d} = 1$ and $N_{A}^{i} = 2$. Here, $N_{e}^{i} = 2$ as the C=C bond contributes two $\pi$ electrons to hyperconjugation and the two atoms (1 and 2) remain connected. Conversely, for the second term in Eq. (17), the two acceptor atoms remain connected, leading to $N_{a} = 1$ and $N_{A}^{j} = 2$. Since the bond bridging the donor and acceptor groups together has been cut (bond between atoms 2 and 3), electrons are no longer being donated to the acceptor, resulting in $N_e^j = 0$.

The functional form of ${\mathcal {S}}$ in Eq. (16) is identical to that of Eq. (14):

18 $$\begin{aligned} {\mathcal {S}}(\Delta _{hyper}) = \frac{1-\exp {(-\lambda \Delta _{hyper})}}{1+\exp {(-\lambda \Delta _{hyper})}} \end{aligned}$$

The parameter $\lambda$ was selected by setting ${\mathcal {S}}(\Delta _{hyper}^{max}) = 0.95$, with $\Delta _{hyper}^{max}$ being the maximum value of $\Delta _{hyper}$ calculated as

19 $$\begin{aligned} \Delta _{hyper}^{max} = \frac{N_e}{N_{A}^{d}} \end{aligned}$$

where $N_e$ is the sum of all the electrons being donated in the hyperconjugated system and $N_{A}^{d}$ is the total number of donor atoms in the system.

The penalty terms discussed thus far all relate to capturing the perturbation in the chemical environment. The following subsections provide the formulation of the penalty terms associated with controlling the fragment size.

***Volume penalty***

The volume penalty ($p_{vol}$) is defined as

20 $$\begin{aligned} p_{vol} = \frac{1}{N_{f}} \sum _k^{N_{f}} \frac{1}{1 + \exp {\left( -14.654 \left( \Delta _{vol}\right) ^2 \right) }} \end{aligned}$$

Here $N_{f}$ is the number of fragments, *k* indexes each fragment, and

21 $$\begin{aligned} \Delta _{vol} = \frac{1}{N_{f}} \sum _k^{N_{f}} \left( \frac{V_k - V_{ref}}{V_{ref}}\right) \end{aligned}$$

where $V_k$ is the volume of fragment *k*, and $V_{ref}$ is the reference volume, which is determined from the target fragment size as discussed further below. The exponent of -14.654 in Eq. (20) was selected based on where the function has sufficiently approached the asymptote value of $p_{vol} = 1$. Specifically, the exponent was selected such that when $|\Delta _{vol}| = 0.5$, $p_{vol}=0.95$.

The volume of a fragment is evaluated according to the following formula

22 $$\begin{aligned} V = \sum _i V_i - \sum _{i<j} V_{ij} \end{aligned}$$

Both *i* and *j* index atoms belonging the fragment. The $V_i$ term is the hard-sphere equivalent atomic volume [28]

23 $$\begin{aligned} V_i = \frac{4}{3}\pi \sigma _i^3 \end{aligned}$$

where $\sigma _i$ is the van der Waals radius of atom *i*, and $V_{ij}$ is the overlapping volume between two atoms [28, 63]

24 $$\begin{aligned} V_{ij} = a_i a_j \exp {\left( -\frac{\alpha _i \alpha _j r_{ij}^2}{\alpha _i + \alpha _j} \right) } \left( \frac{\pi }{\alpha _i + \alpha _j}\right) ^{\frac{3}{2}} \end{aligned}$$

The amplitude $a_i$ is set to a default value of $2\sqrt{2}$, and $r_{ij}$ denotes the distance between atoms *i* and *j*. The $\alpha _i$ term is calculated from $\sigma _i$ as follows

25 $$\begin{aligned} \alpha _i = \pi \left( \frac{3a_i}{4\pi \sigma _i^3}\right) ^\frac{2}{3} \end{aligned}$$

The reference volume $V_{ref}$ is computed using the following equation

26 $$\begin{aligned} V_{ref} = n_t \cdot \frac{1}{N_{A}} \sum _{s \in S} N_{s} V_{s} \end{aligned}$$

Here, $n_t$ represents the target fragment size, while $N_{A}$ denotes the total number of atoms in the molecular system. The set *S* includes all unique atomic elements present in the molecular system, for instance, Argon (Ar), Carbon (C), Nitrogen (N), etc. The variable *s* is used to index these elements. $N_{s}$ indicates the total count of atoms with the symbol *s*, and $V_{s}$ represents the characteristic volume of an atom with symbol *s* in the molecular system, defined as follows

27 $$\begin{aligned} V_s = \frac{4}{3}\pi \sigma _{s}^3 - \frac{1}{\left\| K\right\| } \sum _{i \in K} V_{s,i} \end{aligned}$$

**Fig. 6:**
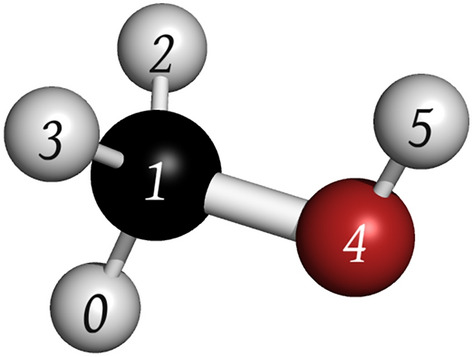
Labelled ball and stick model of methanol. Red, black, white spheres correspond to oxygen, carbon and hydrogen atoms, respectively

In Eq. (27), the first term calculates the hard-sphere volume of an atom denoted by *s*. Here, *K* refers to the set of atoms directly bonded to an atom symbolised by *s*, with *i* indexing these neighboring atoms. $V_{s,i}$ represents the overlapping volume between atom *s* and its neighbors in *K*. Thus, the second term in Eq. (27) averages the overlapping volumes between atom *s* and its adjacent atoms. As an example, consider Fig. 6 illustrating the representative volume of oxygen $V_O$. In this case, the overlapping volumes between two atom pairs (designated as $n_{neigh}^{O} = 2$) are considered: between atoms 1 and 4, and atoms 4 and 5.

***Volume-range penalty***

In the previous discussion on the volume penalty formulation, it is evident that $p_{vol}$ serves as an indicator of the average variation in fragment volumes. This definition implies the possibility of creating a set of fragments with a low $p_{vol}$ value, yet these fragments may vary significantly in size.

To address this issue, we introduce the following volume-range penalty

28 $$\begin{aligned} p_{vrange} = \frac{1}{1 + \exp {(-\lambda (\Delta _{vrange} - d))}} \end{aligned}$$

where *d* is preset to − 0.25, $\lambda$ is adjusted to 11.78, and

29 $$\begin{aligned} \Delta _{vrange} = \frac{V_{range}-V_{ref}}{V_{ref}} \end{aligned}$$

with $V_{ref}$ defined as in Eq. (26), and $V_{range}$ being the difference between the maximum and minimum fragment volumes.

***Number-of-components penalty***

The final component of the scoring function is the penalty term $p_{comp}$, defined as

30 $$\begin{aligned} p_{comp} = \frac{1}{N_{f}} \end{aligned}$$

where $N_{f}$ is the number of fragments the molecular system has been divided into. This penalty term is designed to encourage scenarios with a higher count of fragments, while discouraging situations with fewer fragments. Specifically, $p_{comp}$ seeks to mitigate cases where no fragmentation occurs (*i.e.*, $N_{f} = 1$), which is otherwise favored due to the small $p_{vrange}$ value. In such instances, $\Delta _{vrange} = 0$ and $p_{vrange} \approx 0$, and this effect is counterbalanced by a high $p_{comp}$ value of 1.

***The*** $$\beta _i$$ ***Weights***

Each penalty in Eq. (6) is weighted by a matching $\beta _i$ factor. These factors are constrained to be non-negative ($\beta _i \ge 0$) and subject to a normalisation constraint, ensuring their sum equals one ($\sum _i \beta _i = 1$). The importance of these weights lies in mitigating the impact of double counting. For instance, hyperconjugation and conjugation are interrelated chemical phenomena, and their combined penalties can lead to an over-representation of chemical disturbances due to either conjugation or hyperconjugation. Therefore, the $\beta _i$ weights play a crucial role in moderating the influence of each factor on the overall score, thereby attenuating the effects of potential statistical correlations among penalties. The process of determining the ${\beta _i}$ values involves an optimisation procedure detailed in Sect. 2.4.

#### Representation of fragmentation and solution space

The essence of the automated fragmentation scheme lies in minimising Eq. (6) to obtain an optimal set of fragments, each contributing to the best possible score.

This scheme segments a molecular system into fragments by breaking covalent bonds, resulting in edges being either broken (labelled as ‘1’) or unbroken (labelled as ‘0’). Consequently, a fragmented molecular system is represented as a binary vector, where each element represents an edge in the molecular graph. This binary vector is then used as input for the scoring function in Eq. (6). The resulting score reflects the quality of the corresponding fragmentation.

The objective of the fragmentation algorithm is to partition a molecular system in a way that minimizes the scoring function. This involves an optimisation process, as outlined in Sect. 3.1, which minimises the scoring function and yields the ideal set of fragments.

### Optimisation of scoring function weights

In this subsection, we elaborate on the methodology used for optimising the weights of the scoring function, $\{ \beta _i \}$.

The optimisation of the $\{\beta _i\}$ values, as applied in Eq. (6), is crucial for generating high-quality fragments. These weights quantitatively represent the importance of each penalty term in the scoring function. Suboptimal weightings can lead to an imbalanced scoring function. For instance, excessively high weighting for the volume penalty ($p_{vol}$) might result in an unduly low weight for the potential energy penalty ($p_{pe}$), leading to fragments with significant potential energy variations.

To determine the optimal $\{\beta _i\}$ values, we employed an iterative Bayesian optimisation approach. The weights were fine-tuned using Dataset 1, comprising 800 protein systems with sizes ranging from 108 to 455 atoms.

The Bayesian optimisation aims to minimise the objective function defined as

31 $$\begin{aligned} f = \alpha \frac{1}{n}\sum _i p_{vol}^i + (1-\alpha ) \frac{1}{n}\sum _i {\mathcal {S}}(\Delta E_i) \end{aligned}$$

Here, $p_{vol}$ represents the volume penalty, and $\Delta E$ denotes the energy difference between the total unfragmented system and the MBE2 energy, both computed at the HF/6-31G* theory level. The symbol ${\mathcal {S}}$ in Eq. (31) indicates a normalisation function, analogous to that used for $p_{pe}$, as previously discussed in Sect. 2.3.2, with the exception that the boundary points of the sigmoid function correspond to 1 and 4 kJ $\hbox {mol}^{-1}$. The method for evaluating $p_{vol}$ is also detailed in Sect. 2.3.2. In the equation, *i* indexes each protein system in the dataset, *n* is the total number of protein systems, and $\alpha$ is a hyperparameter. This function, thus, represents a weighted average of the deviations in fragment volume and energy across the dataset. An $\alpha$ value of 0.5 was chosen to balance the significance of volume and energy equally.

The MBE fragmentation method was selected as it forms the basis of other fragmentation methods such as electrostatically-embedded MBE, generalised MBE and FMO.

The design of the objective function in Eq. (31) aims to derive $\{\beta _i\}$ values that guide the scoring function towards producing fragments with minimal MBE2 energy deviations from the complete, unfragmented system, while also maintaining fragment sizes near the desired target. For the optimisation procedure, the target fragment size was set as 50 atoms.

The surrogate model of the objective function was modelled using the Gaussian Process Regressor from the scikit-learn Python package, employing a radial basis function kernel [51]. We set the noise variance and length scales to 1.0. The expected improvement acquisition function guided the selection of subsequent $\{\beta _i\}$ values for sampling.

**Fig. 7:**
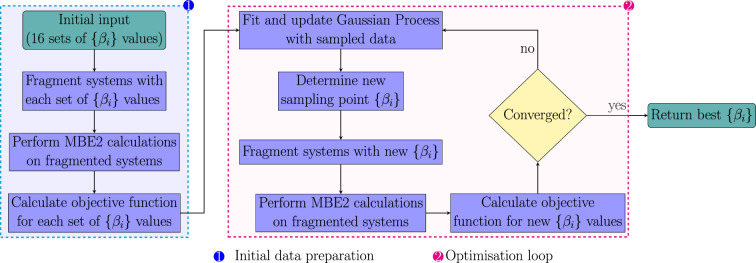
Workflow for the optimisation of $\{\beta _i\}$ values. The numbering corresponds to each of the phases: (1) Initial data preparation; (2) Optimisation loop

Figure 7 illustrates the optimisation workflow. The initial stage involved data preparation to build the Gaussian Process model. This step included computing the objective function *f* (Eq. (31)) for 16 different sets of $\{\beta _i\}$ values. These initial values were derived through a grid search, ranging from 0.1 to 0.9 in 0.1 intervals, as listed in Table 2. As Table 2 indicates, the minimum values for both $\beta _{pe}$ and $\beta _{vol}$ were set at 0.2, reflecting the anticipated higher significance and value of these factors (energy and volume) compared to others.

**Table 2:** Initial datasets of $\{\beta _i\}$ values

$\beta _{pe}$	$\beta _{conj}$	$\beta _{hyper}$	$\beta _{vol}$	$\beta _{comp}$	$\beta _{vrange}$
0.2	0.1	0.1	0.2	0.1	0.3
0.2	0.1	0.1	0.2	0.2	0.2
0.2	0.1	0.1	0.3	0.1	0.2
0.2	0.1	0.1	0.4	0.1	0.1
0.2	0.1	0.2	0.2	0.1	0.2
0.2	0.1	0.2	0.3	0.1	0.1
0.2	0.1	0.3	0.2	0.1	0.1
0.2	0.2	0.1	0.2	0.1	0.2
0.2	0.2	0.1	0.3	0.1	0.1
0.2	0.2	0.2	0.2	0.1	0.1
0.2	0.3	0.1	0.2	0.1	0.1
0.3	0.1	0.1	0.2	0.1	0.2
0.3	0.1	0.1	0.3	0.1	0.1
0.3	0.1	0.2	0.2	0.1	0.1
0.3	0.2	0.1	0.2	0.1	0.1
0.4	0.1	0.1	0.2	0.1	0.1

The second stage in the optimisation of $\{\beta _i\}$ values includes the optimisation loop where each iteration involves updating the Gaussian Process with the recently sampled data, generating the next set of $\{\beta _i\}$ values to sample and using these to fragment and calculate the corresponding MBE2 energies. The objective function is evaluated using the MBE2 energies and volume penalties across all 800 protein systems in Dataset 1. This process was repeated until the minimum objective value remained unchanged for 200 iterations. A total of 645 iterations were performed accordingly.

## Algorithms

This section describes the algorithms employed within QFRAGS for the optimisation of the scoring function (Eq. 6) as well as the overall automated fragmentation algorithm.

### Optimisation of scoring function

#### Restriction of solution space and allowed edges

As previously discussed, each specific solution to the fragmentation problem is represented in the form of a binary vector where each entry corresponds to the state of a bond (1—broken, 0—unbroken). However, since we are aiming for fragments of a specific size, we can eschew from the solution space edges that when severed generate fragments that are too small.

**Fig. 8:**
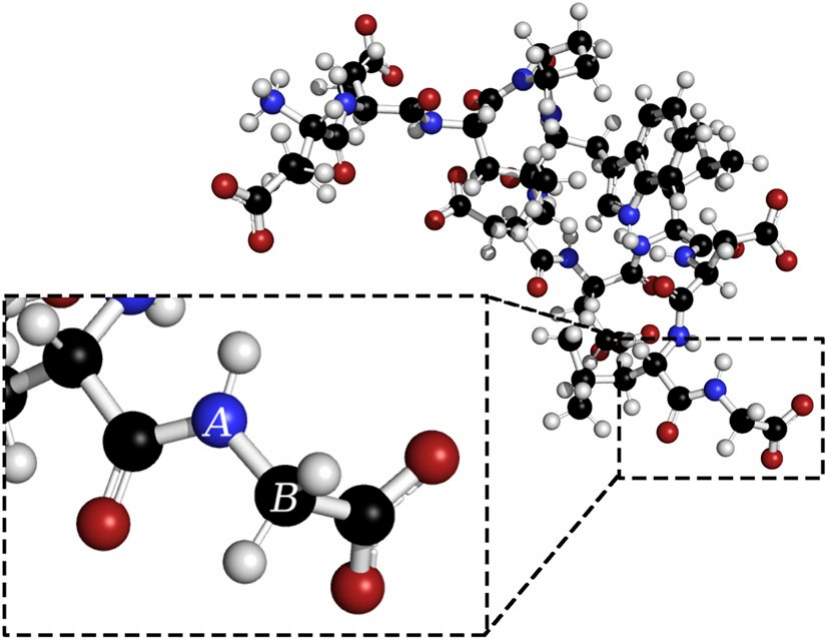
Ball and stick model of MFS-bound Sans CEN2 peptide (PDB ID: 2L7T) displaying an unallowed edge between atoms A and B. See text for more information

To accomplish this, we restrict the solution space to edges that when cut, do not generate fragments that are smaller than 60% of the target fragment size ($n_t$). For example, consider the fragmentation of the protein system consisting of MFS-bound Sans CEN2 peptide, with a PDB ID of 2L7T (174 atoms), into fragments containing $\sim$20 atoms. As shown in Fig. 8 if the A-B edge is cut, two fragments of size 6 and 168 atoms are generated. Since 6 atoms is much smaller than the target size of 20 atoms, we do not consider the edge A-B part of the solution space.

In the present version of the fragmentation code, the solution space is constrained exclusively to single bonds. That is, only bonds with a bond order of one are permitted to be broken. From this point forward, we will refer to the complete collection of edges within the solution space as *allowed edges*. In this implementation, all allowed edges are, without exception, single bonds. Note that according to our definitions conjugated systems can include single bonds and consequently be disrupted by fragmentation.

#### Initial guess

To enhance the optimiser’s capability in identifying optimal solutions, we supply a collection of preliminary approximations. These are instances of fragmentation that represent initial fragment groups.

For formulating these initial guesses, we commence by eliminating all permissible edges from the molecular graph of the system, essentially breaking all bonds within the solution space. This process results in a group of diminutive fragments, which we will call primitive monomers. The fragments constituting the initial guess are subsequently assembled in a recursive manner by combining these primitive monomers, following the methodologies outlined in Algorithms 1 and 2.

**Algorithm 1:**
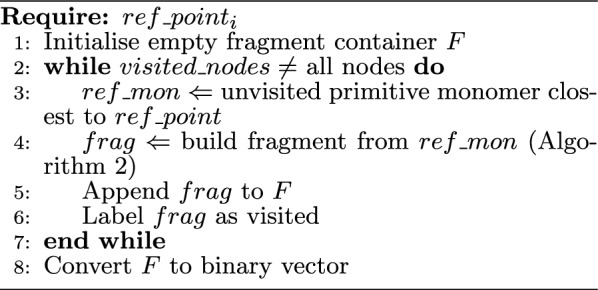
Constructing initial guess for fragmentation instance

**Fig. 9:**
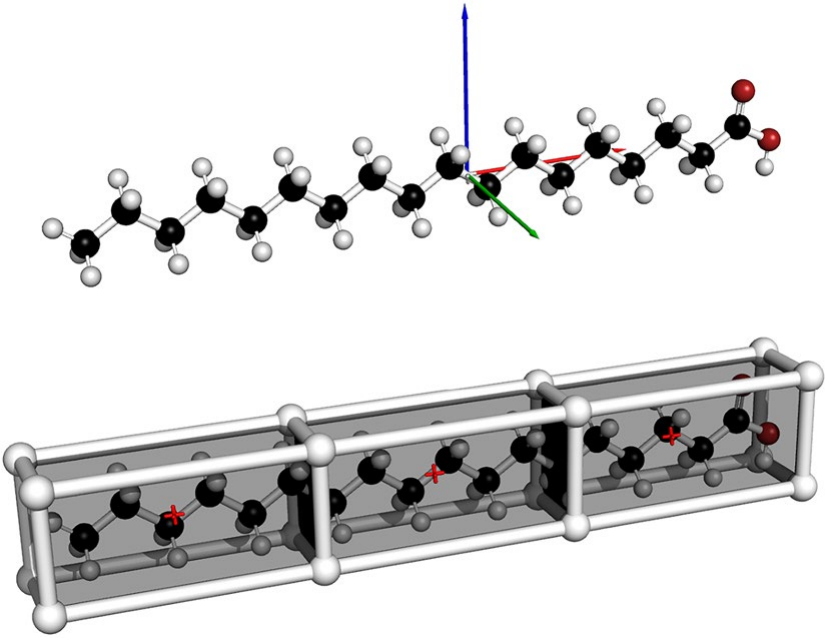
Ball and stick figure of steric acid; *top*: displaying the principal axes of inertia (red, green and blue arrows); *bottom*: displaying the three rectangular intervals formed using the principal axes of inertia, and the reference points (red crosses). All rectangular interval edges are parallel to the principal axes of inertia

In Algorithm 1, we require a reference point $ref\_point_i$ as an input to select a reference primitive monomer ($ref\_mon$) to begin construction of the fragments. The computation of the set of reference points $\{ref\_point_i\}$ is dependent on system size and shape. Specifically, the Euclidean space occupied by the system is partitioned into three-dimensional rectangular intervals along the directions of the principal axes of inertia (the eigenvectors of the inertia tensor), and the midpoint of these intervals are taken as the reference points, as shown in Fig. 9. The inertia tensor was used to ensure that the calculation of the set of $\{ref\_point_i\}$ is invariant to translations, rotations and reflections in the geometry of the structure. Further detail on the computation of the reference points can be found in the Supplementary Information.

Algorithm 1 begins by initialising an empty fragment container which will contain the initial set of fragments obtained with $ref\_point_i$. Lines 2 to 7 describe the strategy of forming these fragments. The algorithm monitors the nodes being visited and continues to build fragments until all nodes have been visited. The construction of each fragment begins at the primitive monomer closest to $ref\_point_i$ that is unvisited (line 3). A fragment, *frag* on line 4, is built from $ref\_mon$ using Algorithm 2. The *frag* object is a collection of primitive monomers, and this fragment is then appended to the fragment container *F*. On line 6, the nodes within *frag* are marked as visited. This process is repeated until all nodes have been visited and the algorithm outputs a binary vector as the initial guess.

Algorithm 2 describes the procedure of constructing a fragment from a reference monomer ($ref\_mon$). Two empty containers are initialised on lines 1 and 2. Both *Q* and *M* hold primitive monomers. However, *Q* represents a queue and *M* is a container that will contain the set of primitive monomers to form a fragment. Next, on lines 3 and 4 $ref\_mon$ is added to both containers. Lines 5 to 16 describe the procedure of constructing the fragment which is a collection of primitive monomers. The algorithm uses a while loop and repeats until *Q* is empty.

Within each iteration of the while loop, *Q* is firstly dequeued and the first primitive monomer in *Q* is assigned to $mon\_v$ (line 6). Following, we iterate across the neighbouring primitive monomers (line 7) of $mon\_v$, where $mon\_w$ denotes the neighbours and the visitation status of each neighbour is checked. If $mon\_w$ has not been visited, it is appended to *Q* and *M* (lines 8 to 10). Next on line 11, the algorithm checks the size (number of atoms) of the growing fragment container *M* and if the size is $\ge$ 90% of the target fragment size ($n_t$), *M* is returned; otherwise the while loop continues. Algorithm 2 repeats until the fragment size condition (line 11) has been satisfied or until the list of neighbouring monomers has been exhausted and *Q* becomes empty.

**Algorithm 2:**
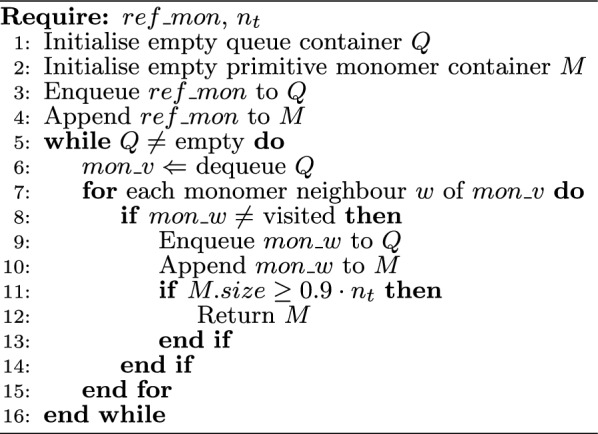
Building fragment from reference monomer

#### Optimiser

The minimisation of the scoring function in Eq. (6) is performed using a genetic algorithm (GA). This was selected as the optimiser for two main reasons. First, it is well suited to exploiting parallel computing architecture at scale, which in turn helps reduce execution time. Second, it is particularly adept at identifying global minima in complex combinatorial challenges [39], such as the fragmentation problem we address, where we aim to find the optimal combination of edges to cut and leave intact.

Within the framework of our GA implementation, each individual within the population represents a distinct fragmentation scenario or a set of fragments. The starting population is comprised of initial guesses, originating from a dataset specifically prepared for this purpose, as discussed in Section 3.1.2.

In the implementation, each gene in an individual corresponds to an allowed edge. As described in Section 3.1.1 concerning the solution space, genes can only take on two possible values: 0 and 1. The total number of parents selected for mating is two if the population size is less than or equal to eight, and it is $\lfloor 0.25 \times$population size$\rfloor$ otherwise. Parents are selected according to the tournament selection technique [58]. The crossover type is a single-point crossover [49] and mutation is random and occurs by replacement.

**Algorithm 3:**
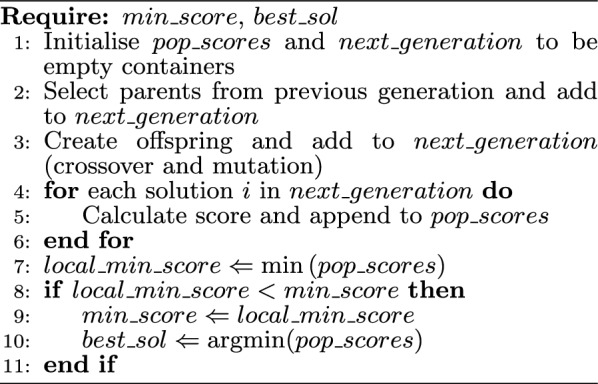
Iteration of the genetic algorithm

The GA approach involves an iterative procedure that aims to explore the solution space by allowing fit individuals to mate and pass its genes to the next generation. Algorithm 3 describes the procedure of creating the next generation in the GA scheme implemented. The fragmentation algorithm monitors the ‘global’ individual ($best\_sol$) with the minimum score ($min\_score$). Fit individuals (parents) are chosen from the previous generation (line 2) and are used to create the offspring for the next generation via crossover (line 3). The solution with the minimum fitness in the next generation ($local\_min\_score$) is compared to $min\_score$, and the global individual and minimum score are updated if a solution with a lower score is found (lines 9 to 10 of Algorithm 3). This iterative process is repeated until either the maximum number of iterations has been reached or if the minimum score has not changed for more than 50 iterations. A maximum number of iterations of 100 was adopted for all our computational experiments.

**Fig. 10:**
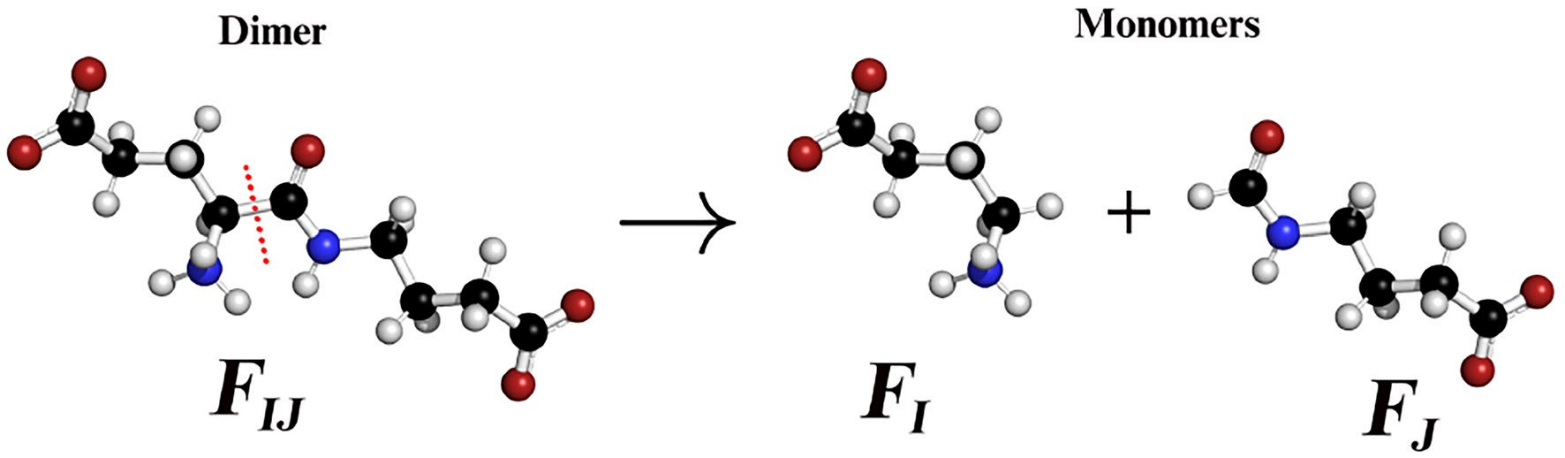
Ball and stick figure of two separate monomers ($F_I$ and $F_J$) and the dimer ($F_{IJ}$) composing these monomers. Atoms coloured black, blue, red and white correspond to carbon, nitrogen, oxygen and hydrogen, respectively. Red broken line denotes a broken bond

To guide the optimiser in locating good quality solutions (low score from Eq. (6)), we utilise the dimer energy (Eq. (32)) to further restrict the solution space throughout the optimisation procedure. The dimer energy correction $\Delta {E}_{IJ}$ is calculated as

32 $$\begin{aligned} \Delta {E}_{IJ} = E_{IJ} - E_{I} - E_{J} \end{aligned}$$

where $E_{IJ}$ represents the energy of the dimer and $E_I$ and $E_J$ are the energies of the monomers. The value of $\Delta {E}_{IJ}$ provides a measure of the energy perturbation when the bond(s) connecting the two monomers in a dimer is/are broken. A force field treatment (UFF) is used for the calculations of the energies ($E_{IJ}$, $E_I$ and $E_J$). As an example consider Fig. 10, where a C-C bond is broken in the dimer ($F_{IJ}$) to produce two monomers ($F_I$ and $F_J$).

If the dimer energy correction corresponding to an edge being cut exceeds a threshold value, this edge is blacklisted and the corresponding bond remains unbreakable in future iterations. The threshold value used is 10 kJ $\hbox {mol}^{-1}$.

The evaluation of the dimer energy correction for each edge being cut is performed within the scoring function calculation for each individual in Algorithm 3. The procedure of blacklisting edges is limited to the first ten iterations of a GA procedure. Otherwise if this continues across the entire optimisation procedure, there is the risk of potentially rendering the set of blacklisted edges to be too large and prevent the optimiser from exploring diverse solutions. By excluding edges associated with large dimer energies from the solution space, we steer the optimiser towards an energetically favourable solution that preserves the integrity of the chemical environment.

### Fragmentation algorithm

Dealing with the complex optimisation challenge of optimally dividing a system into $N_f$ fragments, each with approximately $n_t$ atoms, proves arduous for the optimiser. As molecular systems grow, the difficulty escalates. The optimiser struggles to fragment the system in a feasible number of iterations, hindered by the exponential growth in the combinations of bonds.

To mitigate this issue, we have adopted a recursive fragmentation approach. With this approach, a GA optimiser instance will need to consider a significantly lower number of potential broken bonds at any given time. In turn, breaking a smaller number of bonds results in a smaller cumulative effect on the score, enabling the optimiser to distinguish better between bonds that lead to low and high energy perturbations. Furthermore, the reduction in the problem size that comes with this recursive strategy also means less degenerate solutions for the optimiser to consider.

In the recursive procedure, the molecular system is initially partitioned into *n* larger fragments and each of these fragments is then broken up further. This process is repeated until the fragments are sufficiently close to the target fragment size.

**Fig. 11:**
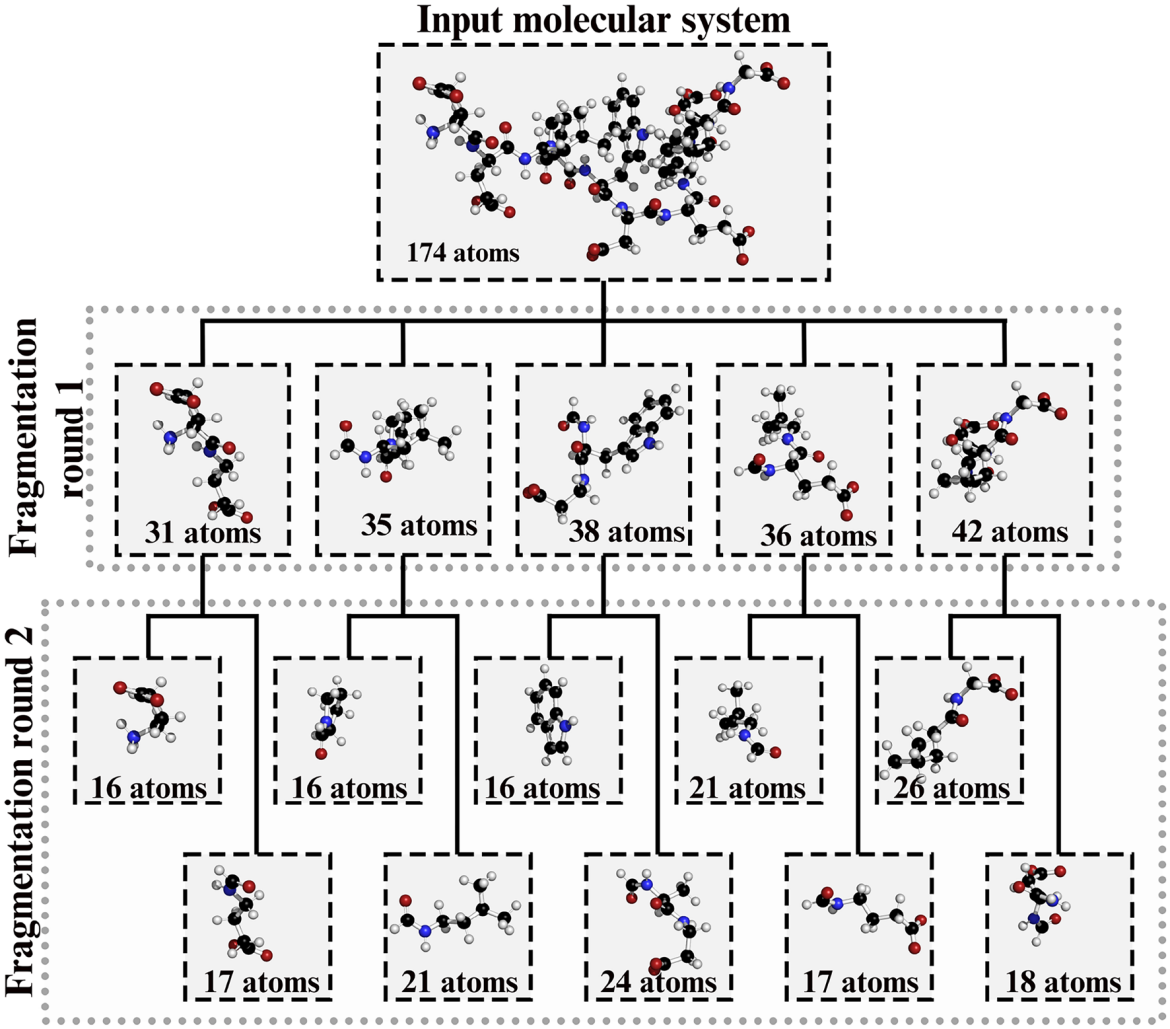
Example of the recursive fragmentation scheme with MFS-bound Sans CEN2 peptide (PDB ID: 2L7T). Target fragment size is 20 atoms. Number of atoms listed for fragments includes hydrogen caps

This recursive procedure is exemplified in Fig. 11, where we fragment a 174-atom protein system (MFS-bound Sans CEN2 peptide, PDB ID: 2L7T), aiming for fragments of approximately 20 atoms. Initially, the algorithm splits the system into five larger fragments of 31, 35, 38, 36, and 42 atoms, respectively. These fragments are then further subdivided to achieve fragments nearing the desired 20-atom size. After two fragmentation stages, ten fragments emerge, each averaging approximately 19 atoms.

**Fig. 12:**
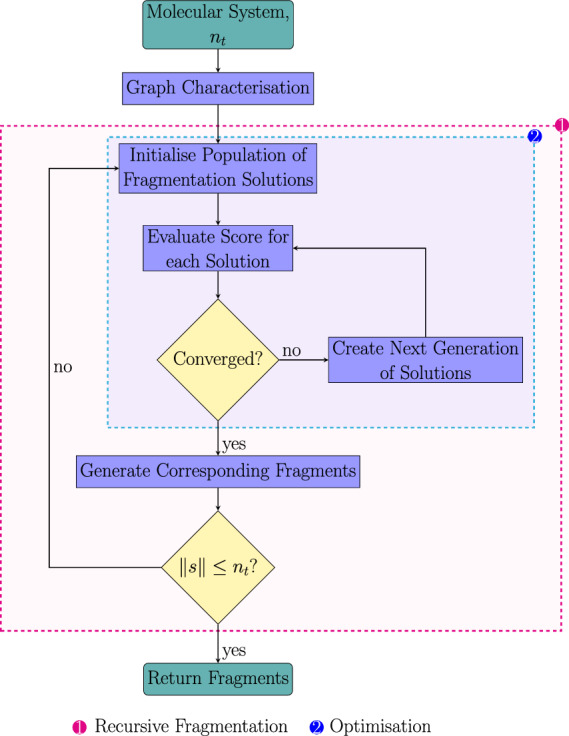
The automatic fragmentation algorithm. The numbering corresponds to different sections of the algorithm: (1) Recursive fragmentation; (2) Genetic algorithm

Figure 12 graphically illustrates the final automatic fragmentation algorithm. The process starts by analysing the molecular system, which involves categorising node and edge attributes and identifying conjugated and hyperconjugated areas. Subsequently, the system undergoes recursive fragmentation, incorporating a series of genetic algorithm optimisation steps. Following each fragmentation phase, the resulting fragment sizes (||*s*||) are assessed against the target size ($n_t$). If a fragment exceeds the target size, it undergoes further recursive fragmentation.

## Results and discussion

### Optimisation of scoring function weights

**Fig. 13:**
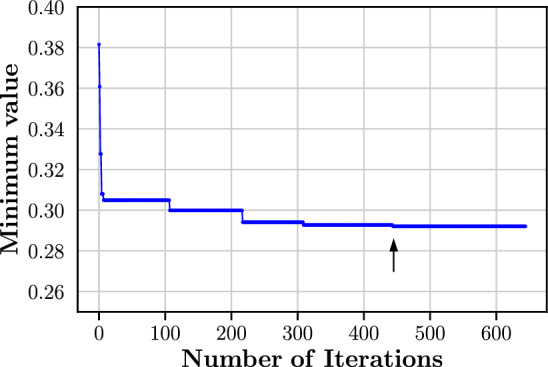
Evolution of the minimum value of *f* (Eq. (31)). Arrow indicates the occurrence of the final minimum value of *f* at iteration number 445

Figure 13 displays the evolution of the minimum value of the objective function *f* (Eq. (31)) over the course of the Bayesian optimisation procedure as described in Sect. 2.4. Within the first five iterations, there is a drastic drop in the minimum value from 0.38152 to 0.30799. Past this, the rate at which the minimum value decreases slows down considerably, indicating a relatively flat optimisation surface. A duration of approximately 100 iterations were required for the occurrence of the next three minimum values; the final minimum value of 0.29205 (see black arrow in Fig. 13 at iteration number 445) occurred 135 iterations after the previous. After 200 iterations of no change in the minimum value, the optimisation procedure was terminated and the corresponding $\{\beta _i\}$ values at iteration number 445 were employed in the fragmentation algorithm.

Table 3 shows the set of $\{\beta _i\}$ values that minimise the scoring function (Eq. 6) against the objective function (Eq. 31).

The term carrying the largest weight in the scoring function is hyperconjugation ($\beta _{hyper} = 0.313325$), which is immediately followed by the volume range ($\beta _{vrange} = 0.294074$). As detailed in Sect. 2.3.2, the six penalty terms in the scoring function are divided into two primary classes: one focusing on maintaining the chemical landscape (encompassing potential energy, conjugation, and hyperconjugation), and the other on managing fragment size (including volume, the number of fragments/components, and volume range). It is noteworthy that the two most heavily weighted factors-hyperconjugation and volume range-belong to these distinct classes. Moreover, the aggregated weights of penalty terms involved in maintaining the chemical environment (0.595084) is greater than that of controlling the fragment size (0.404916). This difference in weight distribution between the two categories implies there is a greater importance to preserving the chemical environment. Later, we show that a good balance between the preservation of the chemical environment and partitioning the system into appropriately sized fragments is been achieved with these weights.

**Table 3:** The set of optimal $\{\beta _i\}$ values obtained from Bayesian optimisation

$\beta _{pe}$	$\beta _{conj}$	$\beta _{hyper}$	$\beta _{vol}$	$\beta _{comp}$	$\beta _{vrange}$
0.135816	0.145943	0.313325	0.109416	0.001426	0.294074

The number of components/fragments exhibits the lowest weight of $\beta _{comp} = 0.001426$. The low weighting for $\beta _{comp}$ is likely influenced by the inclusion of the volume term ($p_{vol}$) in Equation (6). As discussed in Section 2.3.2, the volume penalty term aims to penalise fragmentation instances where the fragments significantly deviate from the desired target size. Similarly, $p_{comp}$ decreases in value as the number of fragments increases, serving a comparable purpose. These factors both encourage fragmentation, but with the presence of $p_{vol}$, the impact of $p_{comp}$ diminishes. The comparative magnitudes of these weights, where $\beta _{vol} = 0.109416$ is larger than $\beta _{comp} = 0.001426$, underscores their primary function of reducing the extent of double-counting.

**Fig. 14:**
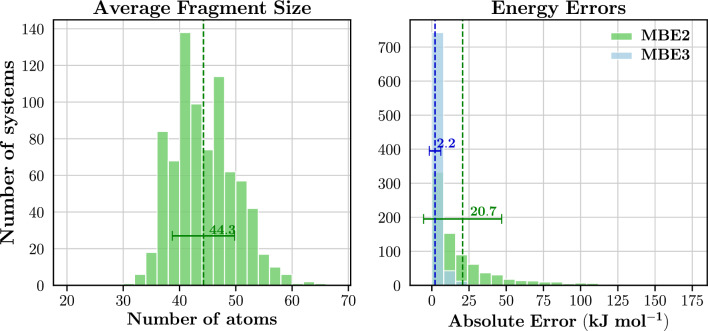
*left*: Distribution of the average fragment size of Dataset 1; *right*: Distribution of absolute energy errors at the MBE2 and MBE3 levels of Dataset 1. All energies were calculated at the HF/6-31G* level of theory. Averages are indicated by vertical lines and the corresponding values are reported. Error bars correspond to one standard deviation

**Table 4:** The set of adjusted optimal $\{\beta _i\}$ values after removal of $\beta _{comp}$

$\beta _{pe}$	$\beta _{conj}$	$\beta _{hyper}$	$\beta _{vol}$	$\beta _{vrange}$
0.136010	0.146151	0.313773	0.109573	0.294494

Due to the small magnitude of $\beta _{comp}$ compared to the other $\{\beta _{i}\}$ values, we performed a two-tailed *t*-test on the set of 800 systems (Dataset 1) to examine its statistical significance. This involved comparing results obtained with $\beta _{comp} = 0.001426$ and $\beta _{comp} = 0$. For $\beta _{comp} = 0$, the remaining $\{\beta _{i}\}$ values were normalised to ensure $\sum _i \beta _i = 1$ (listed in Table 4). In particular, the quantity compared in the *t*-test is similar to Eq. (31), where each molecular system has a fitness value given by

33 $$\begin{aligned} f = \frac{1}{2}p_{vol} + \frac{1}{2}{\mathcal {S}}(\Delta E) \end{aligned}$$

where $p_{vol}$, ${\mathcal {S}}$ and $\Delta E$ are evaluated identical to those in Eq. (31). A *t*-value of 0.014 is obtained and is substantially smaller than the critical *t*-value of 1.961 at the $\alpha = 0.05$ level. Consequently, due to the presence of $\beta _{comp}$ being statistically insignificant at the $\alpha = 0.05$ level, we remove $p_{comp}$ from the scoring function altogether and utilise the weights listed in Table 4 for the remainder of this Article.

Since the weights of the scoring function terms were optimised on Dataset 1, the following text concerns the application of QFRAGS with the optimised $\{\beta _i\}$ values to Dataset 1.

Figure 14 shows the distribution of the average fragment size as well as the energy errors obtained with MBE truncated at the two-body and three-body levels for Dataset 1. The vast majority of systems in Dataset 1 (81.9%) exhibited average fragment sizes ranging between 35 and 50 atoms. Furthermore, 83.0% of systems exhibited average fragment sizes less than the target size of 50 atoms. These resulting fragment sizes are encouraging for our purposes; the standard deviation of 5.5 atoms is relatively small (approximately 10% of the target fragment size) and the majority of the average fragment sizes do not exceed the target fragment size. Exceeding the target fragment size can be problematic due to the growing size of larger fragments (e.g. dimers and trimers) which can lead to memory and convergence issues in fragmentation-based *ab initio* calculations. We will show later in Sect. 4.2.2 that the distribution of the average fragment size narrows with larger system sizes (above 500 atoms).

Regarding the accuracy of total energies, Dataset 1 exhibits relatively low error margins. The mean absolute errors (MAE) are 20.7 and 2.2 kJ $\hbox {mol}^{-1}$ for MBE2 and MBE3, respectively. At the MBE3 level, 84.5% of the systems yielded errors smaller than 4.2 kJ $\hbox {mol}^{-1}$, compared to 29.0% for MBE2. The significant improvement in error rates with MBE3 is expected, as MBE3 accounts for more chemical interactions by incorporating trimers.

The set of $\{\beta _i\}$ values in Table 4 is used in the current implementation of our automated fragmentation algorithm. The subsequent section reports the results obtained from applying QFRAGS to both Dataset 2 and Dataset 3. In the future, we endeavour to expand the application of the fragmentation scheme beyond protein systems, and will re-optimise the weights against a more diverse range of chemical systems.

### Application of QFRAGS

#### Datasets

In this Section, we apply QFRAGS with the optimised $\{\beta _i\}$ values in Table 4 to Datasets 2 and 3. We demonstrate the ability of the automated fragmentation procedure to generate fragment sizes close to the input target fragment size and report on its accuracy by comparison of the single point energies obtained with and without fragmentation. The rationale for employing two distinct test datasets is to examine the impact of system size on energy deviations and fragment dimensions.

#### Fragment size

Figure 3 presents the size distribution of protein systems in Datasets 2 and 3. Dataset 2, with a maximum of 408 atoms, features smaller systems in comparison to Dataset 3, where the largest structure includes 1396 atoms.

**Fig. 15:**
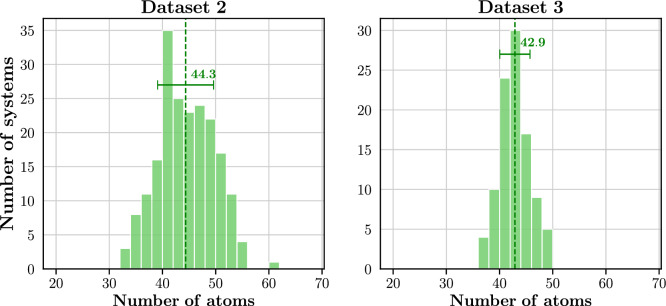
Average fragment size distribution of Dataset 2 and Dataset 3. Averages are indicated by vertical lines and the corresponding values are reported. Error bars correspond to one standard deviation

The distributions of average fragment sizes for Dataset 2 and Dataset 3 are shown in Fig. 15. In Dataset 2, a predominant proportion (81.5%) of systems display mean fragment sizes ranging from 35 to 50 atoms. Conversely, Dataset 3’s distribution is narrower, with 85.0% of its systems having average fragment sizes within the 40 to 50 atom range, compared to only 65.0% in Dataset 2. This variance in distribution patterns is further evident in their standard deviations: Dataset 2 has a higher standard deviation of 5.3 atoms, while Dataset 3’s is 3.2 atoms. These differences are attributable to the recursive fragmentation process and the presence of larger molecular systems in Dataset 3. Given that both datasets aim for a target fragment size of 50 atoms, Dataset 3 undergoes more fragmentation recursions than Dataset 2. Additionally, the larger systems in Dataset 3 offer more possibilities for dividing the system into 50-atom fragments. Consequently, Dataset 3 exhibits a more concentrated distribution, closely aligning with the target fragment size.

For both datasets, there is a very small number of molecular systems that, when fragmented, exhibit mean fragment sizes greater than 50 atoms; this is true for 16.0% and 0.0% of systems in Dataset 2 and Dataset 3, respectively. This was also observed for Dataset 1 and the favourable implications of this were discussed earlier in Section 4.1.

#### Single point energies

Using the fragments produced by QFRAGS, the total energy of molecular systems in Datasets 2 and 3 was calculated at the HF/6-31G* level using the Many-Body Expansion method. The MBE calculations were truncated at the two-body and three-body levels. Subsequently, energies derived from fragmentation were compared with those obtained from full system (unfragmented) calculations at the same HF/6-31G* level.

**Fig. 16:**
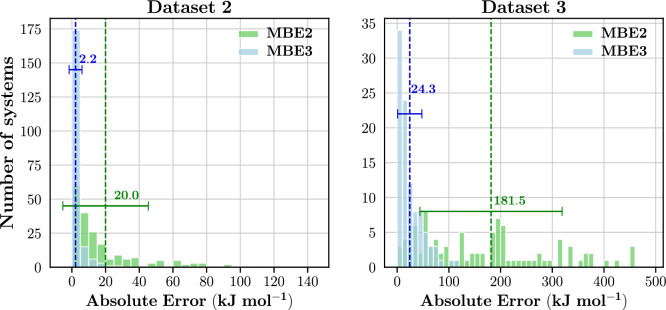
Distribution of absolute energy errors at the MBE2 and MBE3 levels for Dataset 2 and Dataset 3. All energies were calculated at the HF/6-31G* level of theory. Averages are indicated by vertical lines and the corresponding values are reported. Error bars correspond to one standard deviation

Figure 16 presents the distributions of absolute errors at the MBE2 and MBE3 levels. For both datasets, a noticeable reduction in the absolute error is observed as the MBE level increases from dimers to trimers. This reduction is exemplified by the change in MAEs when transitioning from MBE2 to MBE3. Specifically, in Dataset 2, the MAE decreases from 20.0 to 2.2 kJ $\hbox {mol}^{-1}$ and in Dataset 3, the MAE reduces from 181.5 to 24.3 kJ $\hbox {mol}^{-1}$. This improvement in energy accuracy is anticipated and can be attributed to the inclusion of interaction energies in trimers. These findings align with existing literature on hierarchical fragmentation methods [5, 17, 30, 34].

Comparing the two datasets, Dataset 2 exhibits much lower errors than Dataset 3. The MAEs of Dataset 3 are 161.5 and 22.2 kJ $\hbox {mol}^{-1}$ greater than those of Dataset 2 at the MBE2 and MBE3 levels, respectively. At the MBE3 level, 84.5% and 16.0% of systems in Dataset 2 and Dataset 3, respectively, achieved errors less than 4.2 kJ $\hbox {mol}^{-1}$. The higher errors and lower occurrence of accurate results in Dataset 3 are due to the prevalence of larger systems; the average system size in Dataset 3 is 1021 atoms whereas the average system size in Dataset 2 is 277 atoms. The same target fragment size of 50 atoms was used to fragment systems in both datasets. With the systems in Dataset 3 being larger than those in Dataset 2, the number of fragments generated in Dataset 3 will be greater than those in Dataset 2. Correspondingly, more bonds are being broken in the systems belonging to Dataset 3, leading to larger absolute errors.

**Fig. 17:**
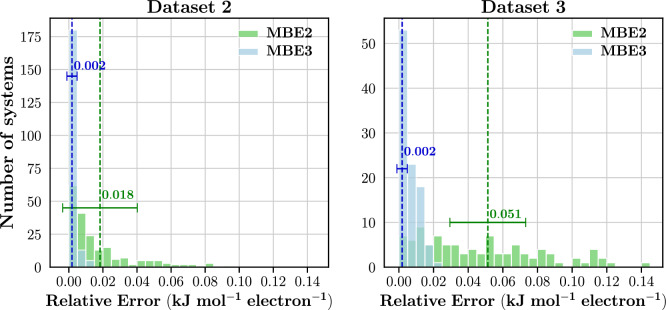
Distribution of relative energy errors at the MBE2 and MBE3 levels for Dataset 2 and Dataset 3. Averages are indicated by vertical lines and the corresponding values are reported. Error bars correspond to one standard deviation. See text for definition of relative errors

To better understand the system size’s impact, we have included the relative error results in Fig. 17. These errors are calculated by dividing the absolute error by the total electron count in the system. Fig. 17 shows the distribution of relative errors for both datasets.

The mean relative error in Dataset 3 at the MBE2 level is 0.051 kJ $\hbox {mol}^{-1}$ per electron compared to the 0.018 kJ $\hbox {mol}^{-1}$ of Dataset 2. Conversely, at the MBE3 level, both datasets exhibit the same relative error of 0.002 kJ $\hbox {mol}^{-1}$ per electron.

Hence, when normalised for system size, at both MBE2 and MBE3 levels, relative errors for the two datasets remain within the same order of magnitude. This contrasts with the absolute errors, where Dataset 3’s MAEs for both MBE2 and MBE3 were consistently larger than those of Dataset 2 by an order of magnitude. These findings suggest that, by considering system size, QFRAGS can achieve comparable relative errors across a broad spectrum of system sizes, ranging from 158 to 1396 atoms.

### Comparison to manual fragmentation

To demonstrate the advantage of the proposed fragmentation scheme, we compare the results of QFRAGS on two samples of 20 protein systems randomly selected from Dataset 1 and Dataset 2 to three manual fragmentation approaches specific to protein systems.

**Fig. 18:**
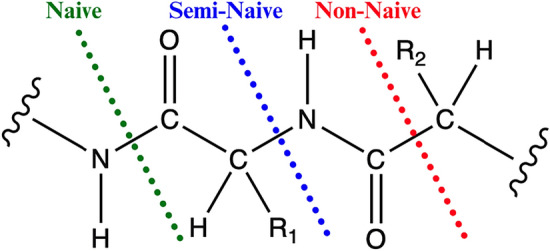
Definition of the three different manual fragmentation schemes employed for the 20 protein systems: naive, semi-naive and non-naive. $\hbox {R}_1$ and $\hbox {R}_2$ denote arbitrary side groups of amino acids

The manual fragmentation approaches will be called naive, semi-naive, and non-naive and corresponding fragments are obtained by severing the C-N amide, $\hbox {C}_\alpha$-N, and $\hbox {C}_\alpha$-C bonds, respectively. Figure 18 illustrates the three different manual fragmentation schemes. These three fragmentation approaches have been explored within literature and it has been consistently demonstrated that the order of increasing accuracy generally follows the order of cutting the C-N amide, $\hbox {C}_\alpha$-N, and $\hbox {C}_\alpha$-C bonds [33, 55, 68].

To control for fragment size, as part of the criteria for bond breaking a fragment size of 50 atoms was selected to match the target fragment size used in QFRAGS.

The sample of 20 structures from Dataset 1 contain systems ranging between 170 and 396 atoms whereas from Dataset 2 the 20 structures comprise between 193 and 400 atoms. The naming of these structures consists of two parts, the first is the PDB code of the original system the system of interest was generated from (see Sect. 2.1). The second part is a subscript which simply indexes the corresponding fragment. For example, 2$\hbox {LTX}_2$ refers to a structure that was obtained from fragmenting the protein system with the PDB code of 2LTX, and the subscript of 2 indexes the second fragment.

**Fig. 19:**
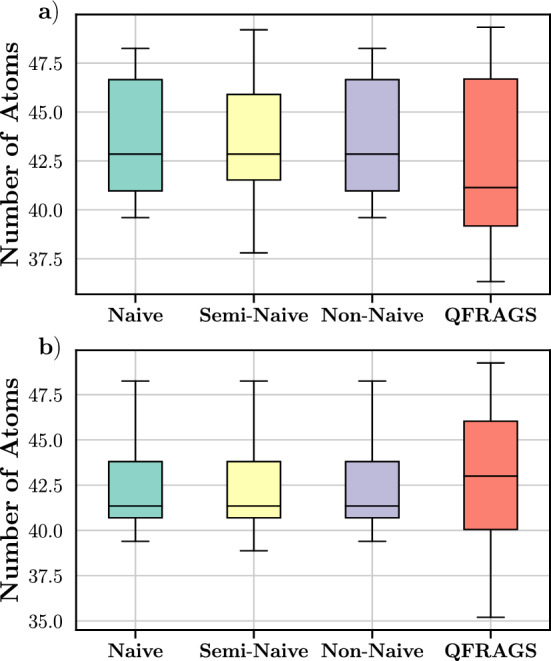
Distribution of average fragment size obtained from fragmenting 20 proteins randomly selected from **a** Dataset 1; and **b** Dataset 2 using the various bond breaking schemes: naive, semi-naive, non-naive and QFRAGS

Figure 19 shows the distribution of average fragment sizes for the two samples of 20 protein systems using the four distinct fragmentation schemes. For the systems from Dataset 1, the naive and non-naive methods yield fragments averaging between 40 and 48 atoms in size. Similarly, the semi-naive method produces fragments with average sizes ranging from 38 to 49 atoms. In contrast, the QFRAGS approach results in fragments averaging between 36 and 50 atoms. The fragment sizes derived from the three manual methods (naive, semi-naive, and non-naive) are more closely aligned with the target size of 50 atoms compared to QFRAGS. A similar outcome can be observed for structures of Dataset 2 where the range of fragment sizes of QFRAGS is larger than those of the manual schemes. Nonetheless, there is a substantial overlap in the average fragment sizes among protein systems across all fragmentation schemes.

**Fig. 20:**
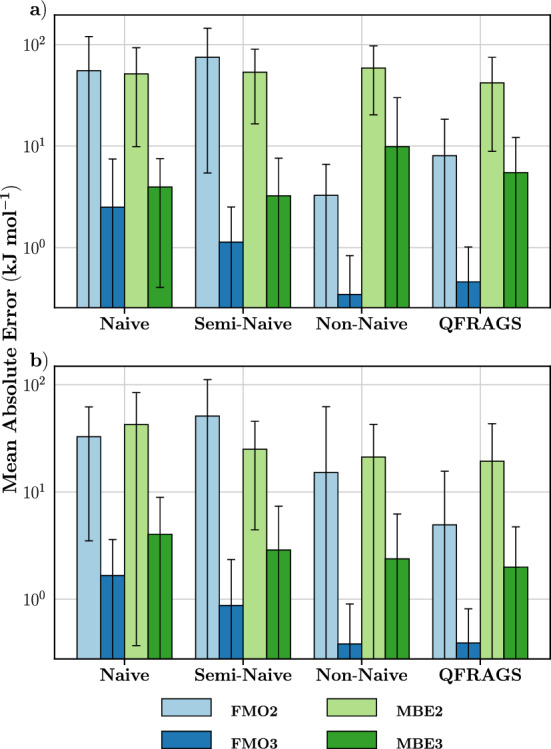
Mean absolute energy errors of various bond breaking schemes (naive, semi-naive, non-naive and QFRAGS) with the fragmentation methods FMO2/3 and MBE2/3 levels across 20 protein systems randomly selected from **a**) Dataset 1; and **b**) Dataset 2. Error bars correspond to one standard deviation. See text for system description of the various bond breaking schemes

Figure 20 shows the mean absolute energy errors calculated using two fragmentation methods (MBE and FMO) at the dimer and trimer levels across the four fragmentation schemes for structures of Dataset 1 and Dataset 2. Across all 40 protein systems, the MBE2 and MBE3 MAEs across the four fragmentation schemes are all within the same order of magnitude. The MBE2 MAEs range between 41.8 and 58.5 kJ $\hbox {mol}^{-1}$, and the MBE3 MAEs range between 3.2 and 9.9 kJ $\hbox {mol}^{-1}$ for systems in Dataset 1. Whilst for Dataset 2, MBE2 MAEs ranged between 19.4 and 42.5 kJ $\hbox {mol}^{-1}$ and MBE3 MAEs ranged between 2.0 and 4.0 kJ $\hbox {mol}^{-1}$.

It should be noted that since the weights of the scoring function were trained on systems belonging to Dataset 1 with MBE, there will be some bias in the MBE results from QFRAGS. It is for this reason that we include systems from Dataset 2. The above results demonstrate the similarity in the behaviour of MBE for systems belonging to and outside of the training set (Dataset 1).

For Dataset 1 structures the FMO errors associated with the non-naive and QFRAGS are consistently an order of magnitude smaller than the naive and semi-naive schemes. Specifically, the FMO2 MAEs of the naive (55.1 kJ $\hbox {mol}^{-1}$) and semi-naive (74.9 kJ $\hbox {mol}^{-1}$) are both an order of magnitude larger than those of the non-naive (3.3 kJ $\hbox {mol}^{-1}$) and QFRAGS (8.5 kJ $\hbox {mol}^{-1}$). This also holds true for FMO3 where the MAEs of the naive (2.5 kJ $\hbox {mol}^{-1}$) and semi-naive (1.1 kJ $\hbox {mol}^{-1}$) fragmentation schemes are greater than those of the non-naive (0.4 kJ $\hbox {mol}^{-1}$) and QFRAGS (0.4 kJ $\hbox {mol}^{-1}$) schemes.

Compared to the FMO results of structures belonging to Dataset 1, those of Dataset 2 contrast in two ways. Firstly, the FMO2 MAEs of all three manual fragmentation schemes are an order of magnitude larger than QFRAGS. Secondly, the FMO3 MAEs of semi-naive, non-naive and QFRAGS are all within the same order of magnitude (less than 1 kJ $\hbox {mol}^{-1}$). However, these differences only occur for the semi- and non-naive manual fragmentation schemes; the behaviour of QFRAGS is consistent across systems in Dataset 1 and Dataset 2.

These differing results between MBE and FMO, specifically, the errors of FMO being generally lower than those of MBE, highlight the importance of the treatment of bond breaking and inclusion of electrostatic potentials in fragmentation methods. MBE fragment calculations do not employ electrostatic potentials and use hydrogen capping to restore valence at the site of broken bonds. The inclusion of hydrogen caps has the potential to perturb the electronic environment and introduce spurious steric effects [67]. Meanwhile, in FMO, fragment energy calculations are performed in the electrostatic potential of surrounding fragments, and furthermore FMO avoids introducing hydrogen caps, and instead the AFO approach is used to treat the broken bonds [38, 47]. The effects of such variability between MBE and FMO are best exemplified through the three-body errors of 2$\hbox {LTX}_2$ fragmented with the non-naive approach, where the MBE3 error (94.2 kJ $\hbox {mol}^{-1}$) is two orders of magnitude larger than that of FMO3 (0.2 kJ $\hbox {mol}^{-1}$).

Furthermore, the MAEs of FMO3 across all four fragmentation schemes are consistently lower than those of MBE3. On the other hand, the FMO2 MAEs are either the same order of magnitude or an order of magnitude less than those of MBE2. Such observations are consistent with the literature on fragmentation methods concerning electrostatic potentials; methods that include electrostatic potentials typically outperform those lacking it [19, 30, 65], albeit being more computationally demanding than the latter.

Furthermore, it is important to recognise that the three manual approaches (naive, semi-naive and non-naive) are suited to protein systems only, whereas QFRAGS possesses no information on the amino acid makeup of the protein systems. On the other hand, the naive, semi-naive and non-naive approaches are specifically tailored to amino acids because these schemes only consider breaking bonds that are found in protein systems. Yet despite this lack of amino acid information, the proposed QFRAGS method is able to achieve MAEs of the same order of magnitude as the three manual fragmentation methods at both the MBE2 and MBE3 levels, outperform the naive and semi-naive schemes with two- and three-body FMO calculations and is comparable to the accuracy of the non-naive scheme with both FMO2 and FMO3.

### Application to glycolipids and lipoglycans

To demonstrate the applicability of QFRAGS beyond protein systems, we applied it to a set of 10 glycolipid and lipoglycan systems ranging between 368 and 727 atoms, with an average of 455 atoms. Unlike proteins, which are composed of well-defined monomeric units (amino acids), these structures lack an intuitive monomeric unit due to the varied lipid component and consequently their manual fragmentation poses a challenging task. Consequently, we selected these structures to analyse the performance of QFRAGS.

**Table 5:** Performance of QFRAGS for glycolipid and lipoglycan systems. MBE2/3 errors are reported in kJ $\hbox {mol}^{-1}$. System name corresponds to the HMDB ID

System	Average	MBE2	MBE3
	Fragment size	Error	Error
0011945	41.8	− 14.6	− 0.2
0011957	40.9	7.8	0.4
0011959	41.2	− 1.3	0.0
0012117	42.3	− 8.6	− 0.2
0012121	47.8	3.7	− 0.1
0012123	47.1	3.0	0.5
0012124	44.5	0.3	− 0.1
0012125	51.8	6.4	− 1.0
0012232	48.7	5.0	0.0
0013470	40.4	27.9	0.1

Table 5 summarises the results of applying QFRAGS to the glycolipid and lipoglycan systems including the average fragment size and MBE2/3 errors. With the exception of one system, the average fragment size is consistently less than the target fragment size of 50 atoms. In fact, the majority of systems (60%) exhibit average fragment sizes ranging between between 40 and 45 atoms.

Concerning the energy errors, the largest MBE2 error (27.9 kJ $\hbox {mol}^{-1}$) belongs to largest system (727 atoms) and this error reduces to 0.1 kJ $\hbox {mol}^{-1}$ with the inclusion of trimer energies. Across the 10 systems, the MAE decreases from 7.9 kJ $\hbox {mol}^{-1}$ at the two-body level to 0.3 kJ $\hbox {mol}^{-1}$ at the three-body level. Once again, this decrease in the MAE is ascribed to the inclusion of interaction energies of trimers. Such results highlight the accuracy of QFRAGS even on systems beyond proteins, the systems where the weights of Eq. (6) were trained on. With the results above, together, these outcomes demonstrate QFRAGS’ capability of generating fragments of a specific size, its accuracy, and its applicability beyond protein systems.

## Conclusion

In this study, we introduced an innovative, automated molecular fragmentation approach, QFRAGS, characterised by its evolutionary optimisation of a scoring function. The proposed approach hinges on three main innovations.

First, the fragmentation process is fully automated, eliminating the need for manual intervention.

Second, traditional energy metrics, which are impractical for large molecular systems, are replaced by a multi-factor scoring function. This function integrates chemical information and implementation aspects, offering a more feasible and effective alternative for fragmenting complex systems.

Third, our approach employs evolutionary strategies to optimise the scoring function. This actively seeks out fragmentation schemes that indirectly minimise energy discrepancies when compared to the energy of the unfragmented, reference system.

The scoring function’s weights were fine-tuned using 800 protein systems, each comprising 108 to 455 atoms. Using the optimised weights, QFRAGS was then applied to over 1000 protein systems with atom counts ranging from 108 to 1396 atoms, targeting fragment sizes of 50 atoms. For systems with less than 500 atoms, the mean fragment sizes achieved with QFRAGS varied between 32 and 65 atoms. In larger systems (505 to 1396 atoms), the average fragment sizes improved, ranging between 37 and 50 atoms. These results show QFRAGS’ efficiency in generating fragments that align well with the desired target size.

Using the fragments generated by QFRAGS, total energies were calculated at the two-body and three-body levels using the Many Body Expansion method, with HF/6-31G* as the theory level. The mean absolute errors for systems less than 500 atoms were 20.6 and 2.2 kJ $\hbox {mol}^{-1}$ at the MBE2 and MBE3 levels, respectively. For larger systems (505 to 1396 atoms), the MAEs increased to 181.5 and 24.3 kJ $\hbox {mol}^{-1}$ at the MBE2 and MBE3 levels, respectively.

Then, a comparison of QFRAGS to three manual fragmentation approaches (naive, semi-naive and non-naive) specific to protein systems was performed on 40 protein structures ranging between 170 and 400 atoms. Total energies were calculated with two fragmentation methods: the Many Body Expansion and the Fragment Molecular Orbital, both at the two-body and three-body levels. All fragmentation strategies generated fragments with similar average sizes close to the target fragment size of 50 atoms. With MBE, the MAEs across QFRAGS and the three manual fragmentation schemes are all within the same order of magnitude. At the MBE2 level, MAEs ranged between 19.4 and 58.5 kJ $\hbox {mol}^{-1}$, meanwhile at MBE3 level, the MAEs fell between 2.0 and 9.9 kJ $\hbox {mol}^{-1}$. On the other hand with the fragment molecular orbital method, the accuracy of QFRAGS (FMO2 and FMO3 MAEs of 6.6 and 0.4 kJ $\hbox {mol}^{-1}$, respectively) are comparable that of the non-naive scheme (FMO2 and FMO3 MAEs of 9.2 and 0.4 kJ $\hbox {mol}^{-1}$, respectively) at both the two- and three-body levels. Both of these schemes are consistently an order of magnitude less than the corresponding MAEs of the naive (FMO2 and FMO3 MAEs of 43.4 and 2.1 kJ $\hbox {mol}^{-1}$, respectively) and semi-naive (FMO2 and FMO3 MAEs of 63.0 and 1.0 kJ $\hbox {mol}^{-1}$, respectively) approaches.

Following, QFRAGS was applied to 10 glycolipid and lipoglycan systems to demonstrate its applicability beyond protein systems and yielded MAEs of 7.9 and 0.3 kJ $\hbox {mol}^{-1}$ at the two- and three-body levels with MBE, respectively.

The results of this study demonstrate the efficacy of the newly proposed automated fragmentation scheme in various aspects. QFRAGS is capable of generating fragments that closely match the desired size. When integrated with MBE and FMO fragmentation methods, it achieves an approximation of the total energy that rivals that of manual, non-naive fragmentation. Furthermore, QFRAGS is generalisable to organic systems beyond proteins. Finally, this study corroborates the importance of employing high-quality fragments and carefully selecting the bonds to be broken in molecular fragmentation approaches.

### Supplementary Information

**Additional file 1.**

## Data Availability

The geometries of the structures in the datasets, geometries of the fragments, full system energies and MBE2/3 energies are available in the GitHub repository.

## Section title of first appendix

An appendix contains supplementary information that is not an essential part of the text itself but which may be helpful in providing a more comprehensive understanding of the research problem or it is information that is too cumbersome to be included in the body of the paper.

## Abbreviations

QFRAGS: Quick fragmentation via automated genetic search
MBE: Many body expansion
FMO: Fragment molecular orbital
MBE1: One-body many body expansion
MBE2: Two-body many body expansion
MBE3: Three-body many body expansion
FMO2: Two-body fragment molecular orbital
FMO3: Three-body fragment molecular orbital
QC: Quantum chemistry
GA: Genetic algorithm
AFO: Adaptive Frozen orbital
MAE: Mean absolute error
