# Supplementary material for: Automatic molecular fragmentation by evolutionary optimisation
Source: J Cheminform. 2024 Aug 19;16:102. doi: 10.1186/s13321-024-00896-z (PMC11331744; doi:10.1186/s13321-024-00896-z)
Supplement: Supplementary file 1 — Additional file 1. [file 13321_2024_896_MOESM1_ESM.pdf]

SUPPLEMENTARY MATERIAL

Fiona C. Y. Yu, Jorge L. Gálvez Vallejo, Giuseppe M. J. Barca

August 7, 2024

Reference points for initial guess

The approach for computing reference points involves dividing the space occupied by the molecular system into three-dimensional rectangular intervals, and the midpoint of each interval is taken as a reference point. The intervals are formed by partitioning in the direction of the three principal axes of inertia. This first involves computing the inertia tensor and diagonalising it. The eigenvectors v_i of the inertia tensor can be arranged in matrix form

$$V = \begin{bmatrix} \vdots & \vdots & \vdots \\ v_1 & v_2 & v_3 \\ \vdots & \vdots & \vdots \end{bmatrix} \tag{1}$$

The coordinates of the atoms are projected from the Euclidean space to the Eigenspace by taking the product of V and the matrix containing the Cartesian coordinates of all atoms as follows

$$V \begin{bmatrix} x_1 & x_2 & \dots & x_n \\ y_1 & y_2 & \dots & y_n \\ z_1 & z_2 & \dots & z_n \end{bmatrix} = \begin{bmatrix} \vdots & \vdots & \vdots \\ v_1 & v_2 & v_3 \\ \vdots & \vdots & \vdots \end{bmatrix} \begin{bmatrix} x_1 & x_2 & \dots & x_n \\ y_1 & y_2 & \dots & y_n \\ z_1 & z_2 & \dots & z_n \end{bmatrix} = \begin{bmatrix} x'_1 & x'_2 & \dots & x'_n \\ y'_1 & y'_2 & \dots & y'_n \\ z'_1 & z'_2 & \dots & z'_n \end{bmatrix} \tag{2}$$

where (x_i, y_i, z_i) and (x'_i, y'_i, z'_i) denote the coordinates of atom i in the Euclidean and Eigenspace, respectively.

In the transformed coordinate system, the range (Δt) along the direction of the three eigenvectors are computed. Δt is given by

$$\Delta t = t_{max} - t_{min} \tag{3}$$

where t_{max} and t_{min} denote the maximum and minimum coordinate values in the Eigenspace along the t-direction. As we have three eigenvectors which form the basis of the Eigenspace, we also have three range values. Of these, we take the minimum range $(\Delta t'_{min})$ to determine the number of intervals (n_{min}) along the direction of the eigenvector that corresponds to $\Delta t'_{min}$. n_{min} is calculated as

$$n_{min} = \left\lceil \frac{\Delta t'_{min}}{\Delta t^*} \right\rceil \tag{4}$$

where Δt^* is a hyperparameter and is given the default length of 15 Å. For the other two directions, the number of intervals (n_{int}) is dependent on the value of n_{min}.

$$n_{int} = n_{min} \left\lceil \frac{\Delta t}{\Delta t'_{min}} \right\rceil \tag{5}$$

Furthermore, the length of each interval along the t-direction (l_t) can be computed according to:

$$l_t = \frac{\Delta t}{n_{int}} \tag{6}$$

For convenience let us denote the set of axes corresponding to the basis vectors (eigenvectors of the inertia tensor) as x', y' and z' axes. The number of intervals along the direction of each of the three basis vectors is $n_{x'}$, $n_{y'}$, $n_{z'}$, and the corresponding interval lengths are $l_{x'}$, $l_{y'}$ and $l_{z'}$, respectively. The total number of intervals (n_{tot}) is given by the following product

$$n_{tot} = n_{x'} n_{y'} n_{z'} \tag{7}$$

The corresponding reference points (in the Eigenspace) is calculated according to the following formula

$$\begin{bmatrix} x' \\ y' \\ z' \end{bmatrix} = \begin{bmatrix} x'_{min} \\ y'_{min} \\ z'_{min} \end{bmatrix} + \frac{1}{2} \begin{bmatrix} l_{x'} \\ l_{y'} \\ l_{z'} \end{bmatrix} + \begin{bmatrix} il_{x'} \\ jl_{y'} \\ kl_{z'} \end{bmatrix} \tag{8}$$

where i, j and k are integer counters along the x', y' and z' directions, respectively, and these take on values between 0 and their corresponding n_{int}.

The reference points calculated with Eq. (8) are located in the Eigenspace. To transform these back to the Euclidean space, we multiply the inverse of V with each of the reference points calculated with Eq. (8). Since V is orthonormal, its inverse is equivalent to its transform. Thus, instead of computing the inverse which is computationally expensive, the reference points in the Euclidean space can be accessed by

$$\begin{bmatrix} x \\ y \\ z \end{bmatrix} = V^T \begin{bmatrix} x' \\ y' \\ z' \end{bmatrix} = \begin{bmatrix} \dots & v_1 & \dots \\ \dots & v_2 & \dots \\ \dots & v_3 & \dots \end{bmatrix} \begin{bmatrix} x' \\ y' \\ z' \end{bmatrix} \tag{9}$$
